# A BINOL-phosphoric acid and metalloporphyrin derived chiral covalent organic framework for enantioselective α-benzylation of aldehydes†

**DOI:** 10.1039/d1sc06045g

**Published:** 2022-01-14

**Authors:** Hui-Chao Ma, Ya-Nan Sun, Gong-Jun Chen, Yu-Bin Dong

**Affiliations:** College of Chemistry, Chemical Engineering and Materials Science, Collaborative Innovation Center of Functionalized Probes for Chemical Imaging in Universities of Shandong, Key Laboratory of Molecular and Nano Probes, Ministry of Education, Shandong Normal University Jinan 250014 P. R. China gongjchen@126.com yubindong@sdnu.edu.cn

## Abstract

The catalytic asymmetric α-benzylation of aldehydes represents a highly valuable reaction for organic synthesis. For example, the generated α-heteroarylmethyl aldehydes, such as (*R*)-2-methyl-3-(pyridin-4-yl)propanal ((*R*)-MPP), are an important class of synthons to access bioactive drugs and natural products. We report herein a new and facile synthetic approach for the asymmetric intermolecular α-benzylation of aldehydes with less sterically hindered alkyl halides using a multifunctional chiral covalent framework (CCOF) catalyst in a heterogeneous way. The integration of chiral BINOL-phosphoric acid and Cu(ii)-porphyrin modules into a single COF framework endows the obtained (*R*)-CuTAPBP-COF with concomitant Brønsted and Lewis acidic sites, robust chiral confinement space, and visible-light induced photothermal conversion. These features allow it to highly promote the intermolecular asymmetric α-benzylation of aldehydes *via* visible-light induced photothermal conversion. Notably, this light-induced thermally driven reaction can effectively proceed under natural sunlight irradiation. In addition, this reaction can be easily extended to a gram-scale level, and its generality is ascertained by asymmetric α-benzylation reactions on various substituted aldehydes and alkyl bromides.

## Abstract 2

We report a new synthetic approach for the intermolecular α-alkylation of aldehydes with alkyl halides based on a BINOL-phosphate and Cu(ii)-porphyrin derived multifunctional CCOF catalyst *via* visible-light induced photothermal conversion.

## Graphical abstract

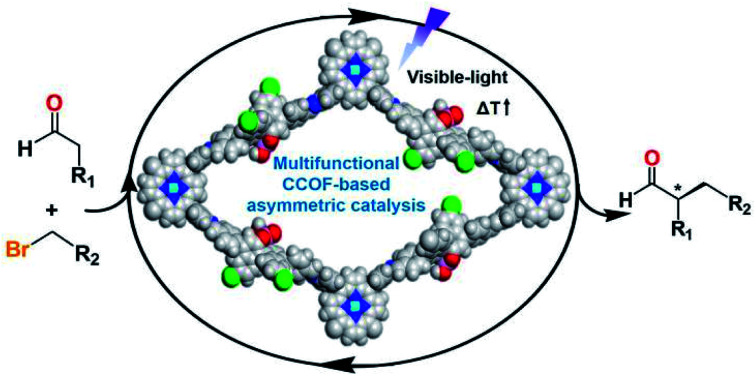

## Introduction

**Scheme 1:**
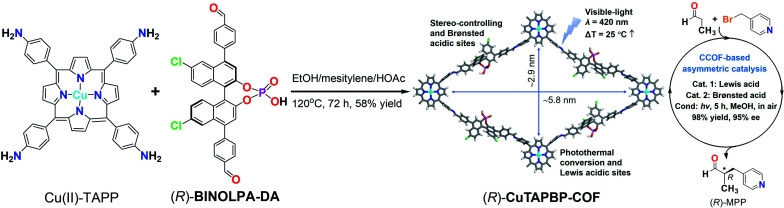
Synthesis and crystal structure of (*R*)-CuTAPBP-COF (left), and its application in enantioselective α-benzylation of aldehydes with an example of (*R*)-MPP synthesis (right).

The catalytic asymmetric α-benzylation of aldehydes, first reported by List and coworkers, has now been recognized as a very valuable reaction for organic synthesis,^1^ and the generated α-heteroarylmethyl aldehydes are an important class of intermediates to access pharmaceuticals and natural products. For example, (*R*)-2-methyl-3-(pyridin-4-yl)propanal ((*R*)-MPP) is a key synthon to synthesize angiogenesis inhibitors, which have been recognized as a bioactive drug candidate for the treatment of tumor proliferation and diabetic retinopathy.^2^ Pioneering work by MacMillan *et al.* has successfully established (*R*)-MPP synthesis based on enantioselective aldehyde α-benzylation *via* dual photoredox organocatalysis.^3^ To our knowledge, no thermally driven, especially solar-powered, (*R*)-MPP synthesis has been reported thus far (Scheme 1).

Although a series of remarkably impressive achievements on the organo-^4^ and transition metal/organo combined catalyzed^5^ intra- and inter-molecular enantioselective α-alkylation of stereohindered α-branched aldehydes have been reported, the simple intermolecular α-alkylation of aldehydes with less sterically hindered alkyl halides remains a huge challenge. The reported methods all involve intramolecular reactions to generate the desired α-benzylation products. Besides, these reported methods, however, still suffered from tedious workup and complicated catalytic systems with disadvantages in recycling and product–catalyst separation. Owing to the increasing awareness of resource, energy and environment issues, the development of convenient, energy- and resource-saving synthetic strategies, especially for the pharmaceutical industry, is highly imperative.

As an attractive class of porous crystalline polymeric materials, covalent organic frameworks (COFs), which were first proposed by Yaghi *et al.* in 2005,^6^ have inherent advantages to be heterogeneous catalysts, especially as chiral heterogeneous catalysts due to their high and regular porosity, robust chiral confinement, extensive functionality, and polymeric nature.^7^

Since the groundbreaking work of Akiyama^8^ and Terada^9^ in 2004, chiral BINOL-derived phosphoric acids, as powerful chiral Brønsted acids, have drawn more and more attention due to their excellent performance in asymmetric catalysis.^10^ On the other hand, metalloporphyrin is not only a type of Lewis acid catalyst,^11^ but also a widely recognized photothermal conversion material that can transfer light energy into thermal energy.^12^ We hypothesized that the incorporation of chiral BINOL-phosphate- and metalloporphyrin-derived monomers into a single COF framework would have several advantages in asymmetric catalysis: first, the well-arranged metalloporphyrin and chiral BINOL-phosphate entities in the CCOF framework not only provide a robust chiral confined space, but also allow Brønsted and Lewis acid derived heterogeneous asymmetric catalysis to be feasible; second, the involved metalloporphyrin can supply the endothermic reaction the needed energy *via* visible-light induced photoconversion, which is significant for the development of solar-powered CCOF-based catalysis; third, the porous CCOF structure provides a commodious pathway for facile diffusion of reaction substrates and products to ensure that the reaction proceeds smoothly. In this multifunctional CCOF design, the chiral confinement space and metalloporphyrin photosensitizer, together with the coexistence of Brønsted and Lewis acidic sites, would enable the asymmetric catalytic reaction to proceed at elevated temperatures with both excellent stereoselectivity and yield.

For achieving α-benzylation of aldehydes in a facile, source- and energy-saving way, we report herein a chiral BINOL-phosphate ((*R*)-BINOLPA-DA) and Cu(ii)-porphyrin (Cu(ii)-TAPP) derived multifunctional CCOF by imine-condensation. Upon visible-light irradiation, the obtained (*R*)-CuTAPBP-COF exhibits excellent catalytic activity and enantioselectivity toward intermolecular α-benzylation of aldehydes with less sterically hindered alkyl halides *via* photothermal conversion (Scheme 1).

## Results and discussion

As shown in Scheme 1, (*R*)-CuTAPBP-COF was prepared as a black-purple crystalline solid by the direct polymerization^13^ of chiral (*R*)-BINOLPA-DA monomer (Fig. S1, ESI†) with Cu(ii)-TAPP *via* imine condensation under solvothermal conditions in 58% yield (EtOH/mesitylene/HOAc, 120 °C, and 72 h). The as-synthesized (*R*)-CuTAPBP-COF was characterized by FT-IR and solid-state CP-MAS spectroscopy, and the existence of BINOL-phosphate and Cu(ii)-porphyrin moieties was fully evidenced (Fig. S2a and b, ESI†). Scanning electron microscopy (SEM) was used to visualize the as-synthesized (*R*)-CuTAPBP-COF, and its blocky particle morphology was observed (Fig. S2c, ESI†). Thermogravimetric analysis (TGA) indicated that (*R*)-CuTAPBP-COF remained intact until temperature over *ca.* 290 °C (Fig. S2d, ESI†), implying its good thermal stability.

**Fig. 1:**
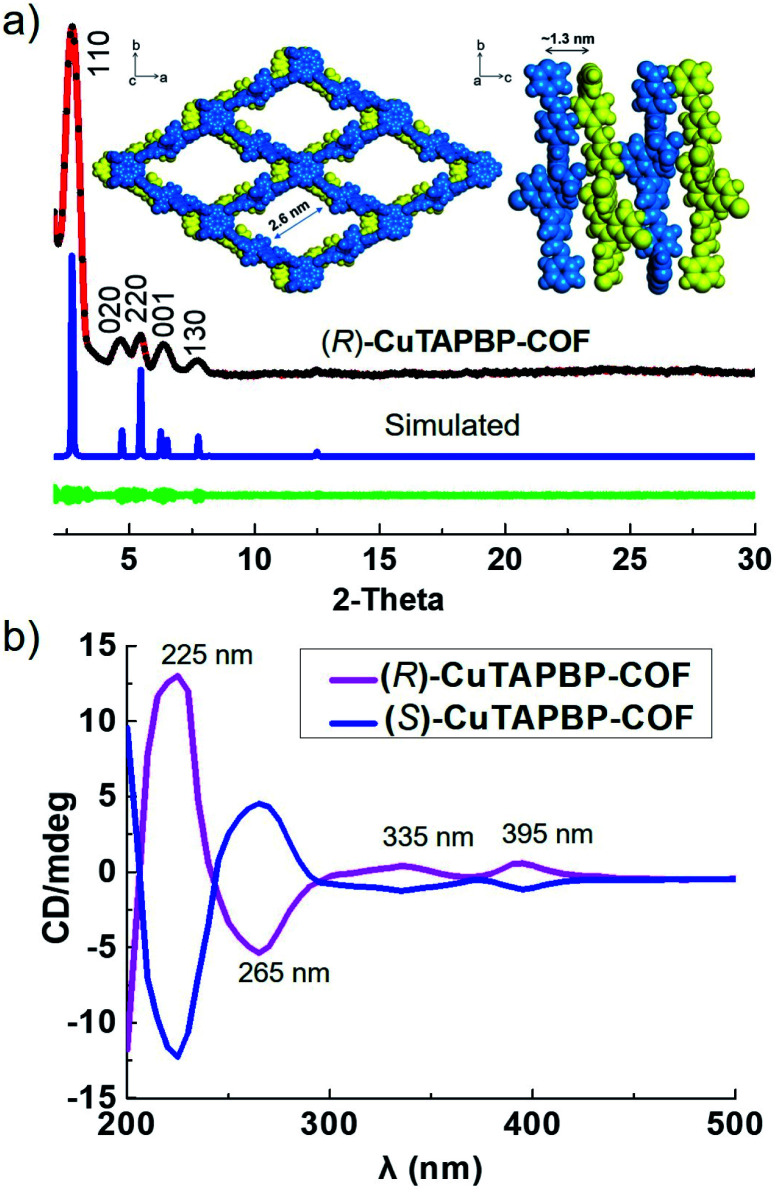
(a) Indexed experimental (red), Pawley-refined (black), and simulated (blue) PXRD patterns of (*R*)-CuTAPBP-COF. The difference plot is presented in green. Top and side views of its simulated crystal structure are shown in insets. (b) CD spectra showing that the pairs of (*R*)- and (*S*)-CuTAPBP-COF are mirror images of each other.

As revealed by its powder X-ray diffraction (PXRD) pattern (Fig. 1a), (*R*)-CuTAPBP-COF was obtained as a highly crystalline material. Structural modeling was thus conducted with Materials Studio software (ver. 2018).^14^ The most probable structure of (*R*)-CuTAPBP-COF was simulated, analogous to that of (*R*)-CuTAPBP-COF, as a 2D staggered layered-net using the chiral space group of *C*_2_ with the optimized parameters of *a* = 66.38 Å, *b* = 37.65 Å, *c* = 14.61 Å, and *β* = 104.46° (residual w*R*_p_ = 2.04% and *R*_p_ = 2.01%, Table S1†). As indicated in Fig. 1a, the intense PXRD peaks at 2.7, 4.7, 5.4, 6.5 and 7.7° displayed by (*R*)-CuTAPBP-COF respectively correspond to the (110), (020), (220), (001) and (130) planes. The structural modeling shows that Cu(ii)-TAPP is linked together *via* chiral BINOL-phosphate into a 2D layer extended in the crystallographic *ab* plane with a rhombus cavity, in which the diagonal C⋯C distance is *ca.* 2.9 and 5.8 nm, respectively (Scheme 1). These layers further stack together in an AB fashion (interlayer Cu(ii)⋯Cu(ii) distance of *ca.* 1.3 nm) to generate rhombus channels with a reduced pore size (The shortest opposite C⋯C distance of *ca.* 2.7 nm) due to their staggered stacking mode (Fig. 1a). The transmission electron microscopy (TEM) image further confirmed that (*R*)-CuTAPBP-COF is highly crystalline (Fig. S2e, ESI†). Notably, (*R*)-CuTAPBP-COF herein, with other types of possible simulated space groups, gave a PXRD pattern that significantly deviated from the measured profile (Fig. S2f, ESI†).

The permanent porosity of (*R*)-CuTAPBP-COF was proved by gas adsorption–desorption measurements. As is shown, the N_2_ absorption amount of (*R*)-CuTAPBP-COF at 77 K is 339.1 cm^3^ g^−1^, and its corresponding surface area calculated on the basis of the BET model is 1002.5 m^2^ g^−1^ (Fig. S3, ESI†). The pore size distribution curve, recorded by nonlocal density functional theory (NLDFT) analysis, indicated that (*R*)-CuTAPBP-COF possessed a narrow pore diameter distribution centered at *ca.* 2.6 nm (Fig. S3 inset, ESI†), which is well in accord with its simulated crystal structure.

The intrinsic chiral nature of (*R*)-CuTAPBP-COF was verified by its circular dichroism (CD) spectrum. As indicated in Fig. 1b, (*R*)-CuTAPBP-COF is optically active and showed a positive Cotton effect at 225, 335 and 395 nm and a negative dichroic signal at 265 nm.

With (*R*)-CuTAPBP-COF in hand, we then examined its asymmetric catalytic activity based on the model reaction of propionaldehyde with 4-(bromomethyl)pyridine to form (*R*)-MPP, a synthon to angiogenesis inhibitors. As shown in Table 1, catalytic reactions were performed under different conditions, including different possible solvents, bases, and catalyst amounts with or without visible-light irradiation. The best result was observed when the reaction was conducted in MeOH (1.5 mL) under 420 nm (2.5 W cm^−2^) light irradiation for 5 h in the presence of (*R*)-CuTAPBP-COF (10 mg, 0.17 mol%, 1.8 mol% Cu and 1.7 mol% phosphate equiv.) and 2,6-lutidine (HBr scavenger) to afford (*R*)-MPP in 98% yield with 95% ee (TON = 54.5 and TOF = 10.9 h^−1^) (Table 1, entry 1). The measured reaction system temperature was up to *ca.* 50 °C, suggesting that (*R*)-CuTAPBP-COF enables efficient visible-light triggered photothermal conversion even at a catalytic amount. Compared to MeOH, other possible solvents such as toluene and MeCN did not give satisfactory catalytic results (Table 1, entries 2 and 3). In the absence of 2,6-lutidine (Table 1, entry 4) or replacing it with a neutral acid (propylene oxide) (Table 1, entry 5), slightly lower yields (95 and 92%) and ee values (95 and 91%) were obtained. In addition, more catalyst loading (0.25 mol%) could not further enhance the yield and enantioselectivity (Table 1, entry 6), while with less catalyst loading, 0.09 mol% instead of 0.17 mol%, the product was isolated in a lower 85% yield with 93% ee under the given conditions (Table 1, entry 7).

**Table 1:** Optimization of (*R*)-CuTAPBP-COF-catalysed synthesis of (*R*)-MPP by enantioselective α-benzylation of aldehydes *via* photothermal conversion^a^

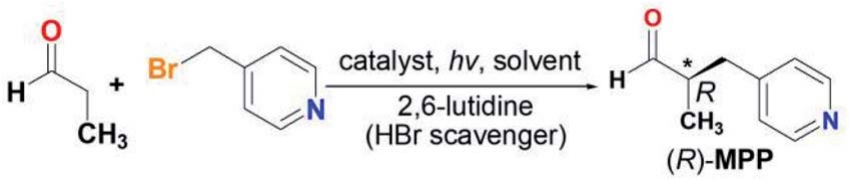				
Entry	Catalyst	Solvent	*T* (°C)/*hv*	Yield (ee)^b^ (%)
1	(*R*)-CuTAPBP-COF	CH_3_OH	r.t./*hv*	98 (95)
	(0.17 mol%)			
2	(*R*)-CuTAPBP-COF	PhMe	r.t./*hv*	22 (86)
	(0.17 mol%)			
3	(*R*)-CuTAPBP-COF	CH_3_CN	r.t./*hv*	56 (90)
	(0.17 mol%)			
4^c^	(*R*)-CuTAPBP-COF	CH_3_OH	r.t./*hv*	95 (95)
	(0.17 mol%)			
5^d^	(*R*)-CuTAPBP-COF	CH_3_OH	r.t./*hv*	92 (91)
	(0.17 mol%)			
6	(*R*)-CuTAPBP-COF	CH_3_OH	r.t./*hv*	98 (94)
	(0.25 mol%)			
7	(*R*)-CuTAPBP-COF	CH_3_OH	r.t./*hv*	85 (93)
	(0.09 mol%)			
8	(*R*)-CuTAPBP-COF	CH_3_OH	50 °C/dark	98 (92)
	(0.17 mol%)			
9	(*R*)-CuTAPBP-COF	CH_3_OH	r.t./dark	34 (88)
	(0.17 mol%)			
10	(*R*)-TAPBP-COF	CH_3_OH	r.t./*hv*	41 (92)
	(0.17 mol%)			
11	(*R*)-TAPBP-COF	CH_3_OH	50 °C/dark	43 (91)
	(0.17 mol%)			
12	Cu-TAPP monomer	CH_3_OH	r.t./*hv*	62 (−)
	(1.8 mol Cu%)			
13	Cu(OAc)_2_	CH_3_OH	r.t./*hv*	57 (−)
	(1.8 mol Cu%)			
14	(*R*)-BINOLPA-DA	CH_3_OH	r.t./*hv*	31 (30)
	(1.7 mol P%)			
15	H_3_PO_4_	CH_3_OH	r.t./*hv*	32 (−)
	(1.7 mol P%)			
16^e^	Cu-TAPP/(*R*)-BINOLPA-DA	CH_3_OH	r.t./*hv*	95 (33)
17	(*R*)-CuTAPBP-COF	CH_3_OH	r.t./sunlight	65 (94)
	(0.17 mol%)			

a Reaction conditions: catalyst, propanal (79 µL, 0.5 mmol), 4-(bromomethyl)pyridine (86 mg, 0.5 mmol), 2,6-lutidine (88 µL, 0.75 mmol), CH_3_OH (1.5 mL), 300 W xenon with a power density of 2.5 W cm^−2^ (*λ* = 420 nm), and 5 h, in air.

b Product structure was determined by ^1^H NMR and MS spectroscopy, yields were determined by GC on a HP-5 column, and ee values were determined by HPLC with a Chiralcel OD-H column (95 : 5 = *n*-hexane : isopropanol, 1.0 mL min^−1^, and 254 nm) (Fig. S4, ESI).

c The reaction was performed in the absence of 2,6-lutidine.

d The reaction was performed in the presence of propylene oxide.

e Mixture of Cu(ii)-TAPP (1.8 mol% Cu) and (*R*)-BINOLPA-DA (1.7 mol% P) with a molar ratio of 1 : 2.

It is worth noting that when the reaction was carried out at 50 °C in the dark, the product was still generated in 98% yield (92% ee, Table 1, entry 8), while only 34% yield (88% ee) was obtained when the reaction was conducted in the dark at room temperature (Table 1, entry 9). Therefore, the (*R*)-MPP synthesis herein is a typical thermally driven process rather than a photochemical reaction. Notably, when copper-free (*R*)-TAPBP-COF was used to perform the reaction under light-irradiation or heating conditions, the reaction afforded moderate yields (41 or 43%) but with excellent enantioselectivity (92 or 91% ee) (Table 1, entries 10 and 11). Furthermore, with the aid of Cu(ii)-TAPP monomer or Cu(OAc)_2_, the product was generated in 62% or 57% yield without any enantiomeric excess (Table 1, entries 12 and 13). Also, in the cases of (*R*)-BINOLPA-DA monomer and H_3_PO_4_, the desired product was respectively obtained in 31% yield with 30% ee and 32% ee without any enantiomeric excess (Table 1, entries 14 and 15). These obtained results proved that both Cu(ii) (Lewis acid) and phosphate (Brønsted acid) are the catalytic sites in the reaction, while the (*R*)-BINOL entity shapes the COF, which then controls the enantioselectivity. When the reaction was carried out with Cu(ii)-TAPP and (*R*)-BINOLPA-DA monomers at a molar ratio of 1 : 2 under light irradiation (Table 1, entry 16), the desired product was obtained in 95% yield but with only a 33% ee value, indicating that the chiral confined space of the CCOF possessed a much more powerful chiral templating effect than the corresponding chiral monomer at elevated temperature. Thus, the CCOF herein not only ensured a high reaction yield, but also permitted a high enantiopurity at elevated temperature. This CCOF-based asymmetric catalytic approach significantly overcame the conventional drawbacks associated with some low-yield asymmetric catalytic reactions caused by the low-temperature conditions.

What is particularly interesting is that this light-induced thermally driven α-benzylation of aldehydes could effectively proceed under natural sunlight irradiation. As shown in Table 1 (entry 17), the reaction system temperature increased to *ca.* 46 °C, and (*R*)-MPP was obtained in 65% yield with 94% ee in 5 h upon natural sunlight irradiation. This is a beneficial trial on the CCOF-based window ledge asymmetric reaction.

**Fig. 2:**
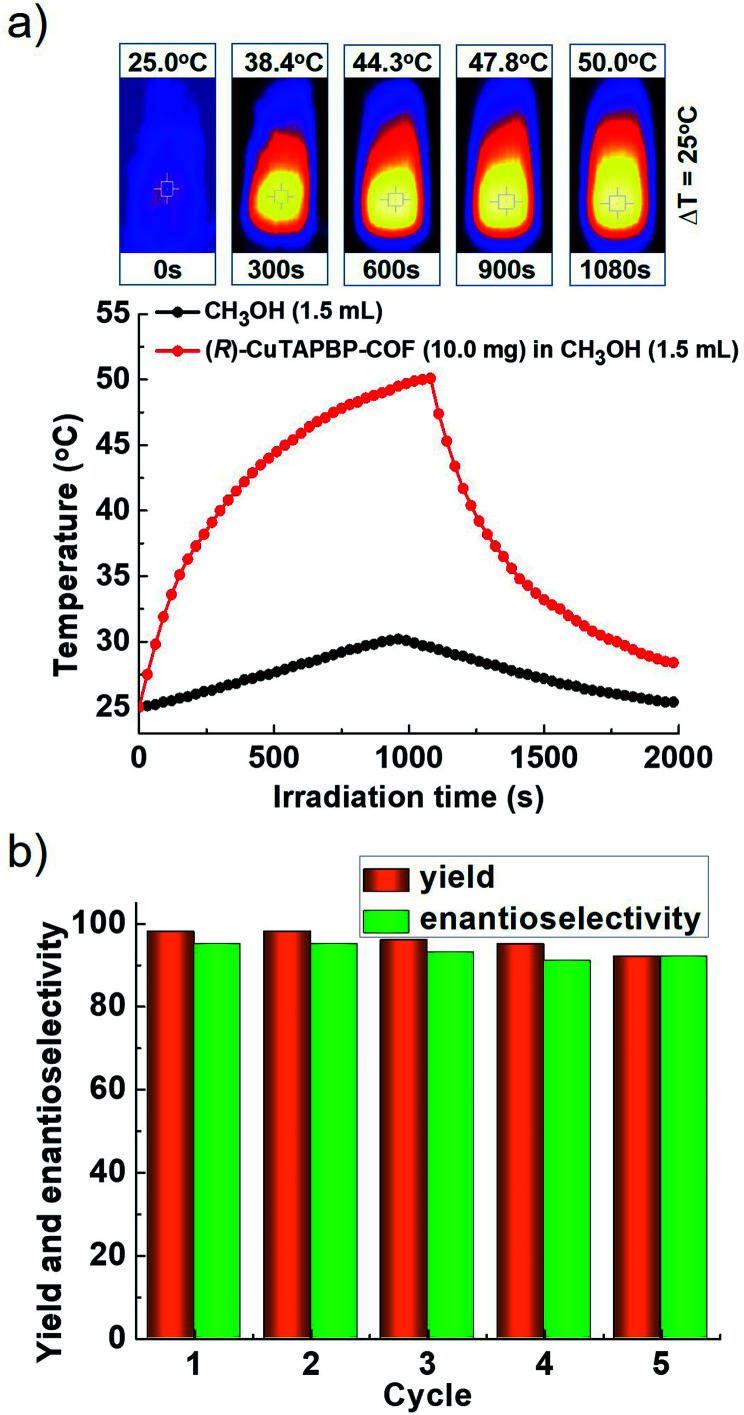
(a) Photothermal behaviour of (*R*)-CuTAPBP-COF in MeOH (1.5 mL). Visible-light source: 300 W xenon lamp, and *λ* = 420 nm with a power intensity at 2.5 W cm^−2^. (b) Bar-shaped graph of the recycling test for (*R*)-CuTAPBP-COF-catalysed (*R*)-MPP synthesis under the given conditions.

For further understanding the photothermal conversion behaviour of (*R*)-CuTAPBP-COF, the visible-light induced temperature increase (Δ*T*) of the above reaction system (*i.e.*, MeOH) in the presence of a catalytic amount of (*R*)-CuTAPBP-COF was examined in terms of its absorption spectrum (Fig. S5a, ESI†). When (*R*)-CuTAPBP-COF (10 mg) in MeOH (1.5 mL) was irradiated with visible light at 420 nm for 18 min, a significant temperature increase (Δ*T*) of 25 °C was observed (Fig. 2a), which is well consistent with the observation in its catalytic experiments (Table 1).

The heterogeneous catalytic nature of (*R*)-CuTAPBP-COF was evidenced by a leaching experiment (Fig. S5b, ESI†), and it can be reused at least five times under the given conditions. As indicated in Fig. 2b, the yield of (*R*)-MPP is still up to 92% after five catalytic cycles (Fig. S5c, ESI†), but its crystallinity and structural integrity were intact (Fig. S5d, ESI†).

Although the mechanism of this reaction is still under investigation, the participation of Lewis (Cu(ii)) and Brønsted (phosphate) acids may activate the aldehyde before the benzylation. And then, the first-formed heterodimer between the aldehyde and Cu-TAPP and (*R*)-BINOLPA further reacts with the radical species generated from alkyl halides to afford the final α-benzylation product under the given conditions (Fig. S6, ESI†). To gain more insight into the mechanism, a radical clock experiment was also conducted. When cyclopropanecarboxaldehyde (a radical clock compound) and 4-(bromomethyl)pyridine were subjected to either visible-light irradiation or heating conditions, a ring-opening product was generated, further verifying the existence of the radical species during the reaction process (Fig. S7, ESI†).

Besides, the gram-scale preparation of (*R*)-MPP was also carried out. Gratifyingly, the enantioselective α-benzylation of propanal proceeded very smoothly upon visible-light irradiation and afforded (*R*)-MPP in 93% yield (1.21 g) with 93% ee within 5 h (Fig. S8, ESI†), which provides a strong foundation for the future development of CCOF-based fine chemical production *via* photothermal conversion.

The scope of (*R*)-CuTAPBP-COF-catalysed enantioselective α-benzylation of aldehydes was also investigated utilizing various substrates (Table 2). Different aldehydes and alkyl bromides provided excellent yields (91–98%, Table 2, entries 1–8) with excellent ee values (91–94%, Table 2, entries 1–8), except for those large-sized substrates (Table 2, entries 9–10), implying that this asymmetric α-benzylation of aldehydes herein is a typical internal surface catalysis (Fig. S9 and S10, ESI†).

**Table 2:** Scope of the (*R*)-CuTAPBP-COF-catalysed enantioselective α-benzylation of aldehydes^a^

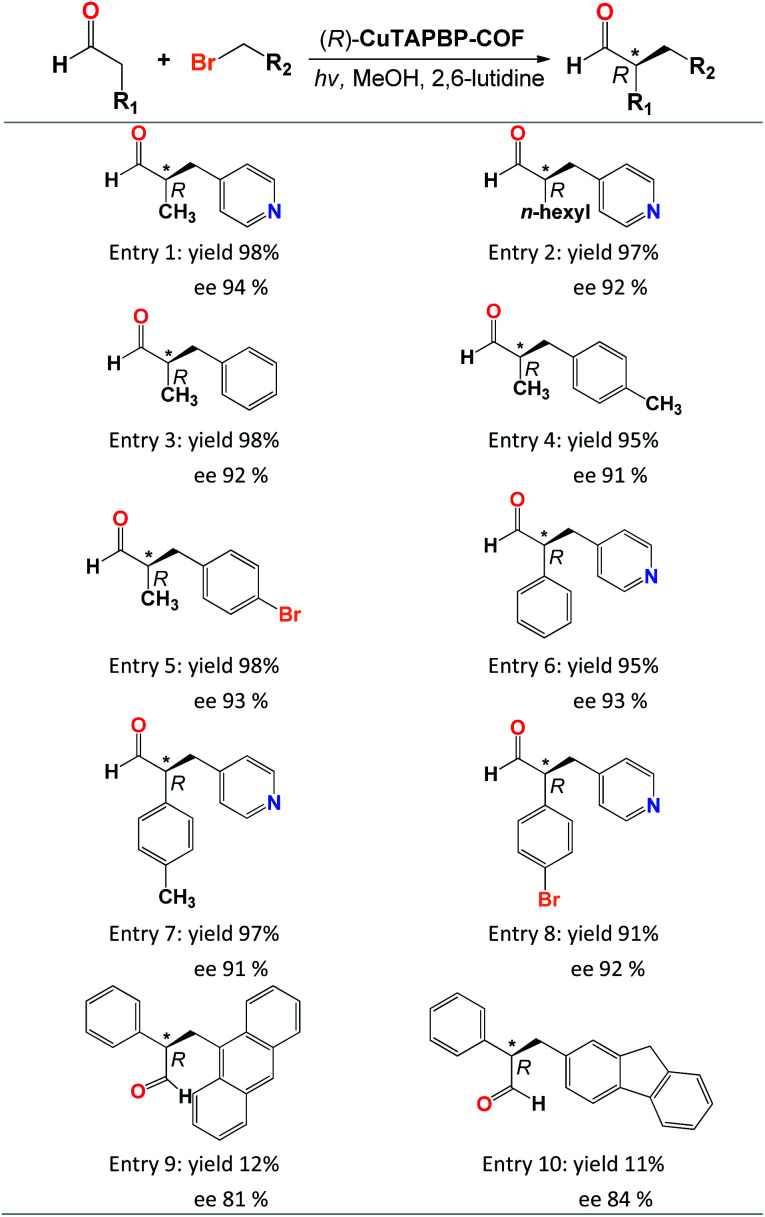

a Reaction conditions: (*R*)-CuTAPBP-COF (10 mg, 0.17 mol%), aldehydes (0.5 mmol), alkyl bromides (0.5 mmol), 2,6-lutidine (0.75 mmol), CH_3_OH (1.5 mL), 300 W xenon with a power density of 2.5 W cm^−2^ (*λ* = 420 nm), and 5 h, in air. The product structure was determined by ^1^H NMR and MS spectroscopy, the yield was determined by GC on a HP-5 column, and ee values were determined by HPLC with a Chiralcel OD-H column (95 : 5 = *n*-hexane : isopropanol, 1.0 mL min^−1^, and 254 nm).

As shown above, the merger of catalysis with the chiral templating effect of (*R*)-CuTAPBP-COF resulted in not only high yield but also excellent enantioselectivity. For further understanding this subtle chiral induction, we synthesized (*S*)-CuTAPBP-COF (Fig. 1b and S11, ESI†), and moreover, performed the (*S*)-CuTAPBP-COF-catalysed α-benzylation of propanal. As expected, the reaction afforded (*S*)-MPP in 98% yield with 94% ee (Fig. S12, ESI†). This implies that we can subjectively manipulate the product chirality as needed by tuning the CCOF chirality,^15^ which would be very significant for the synthesis of optically pure drugs with the same composition but with opposite chirality along with different medical functionalities.

## Conclusions

In summary, we developed a new asymmetric synthetic approach for the intermolecular enantioselective α-benzylation of aldehydes with less sterically hindered alkyl halides based on a multifunctional CCOF catalyst in a heterogeneous way. The CCOF with chiral BINOL-phosphoric acid and Cu-porphyrin species not only can activate the substrates,^16^ but also can induce the substrates to form the product with high optical purity. In addition, the needed energy for this endothermic α-benzylation reaction herein is supplied by visible-light induced photothermal conversion. We believe that our multifunctional CCOF-based asymmetric approach is a general and powerful enabling synthetic methodology, and moreover, can be widely used for the preparation of many other kinds of chiral fine chemicals in an eco-friendly, energy- and resource-saving way.

## Data availability

All the data have been included in the ESI.†

## Author contributions

All authors contributed extensively to the work presented in this paper. Y.-B. Dong and G.-J. Chen conceived the research project. H.-C. Ma performed the experiments and the data analyses. Y.-N. Sun assisted with the data collection. Y.-B. Dong wrote the manuscript with the input from the other authors.

## Conflicts of interest

There are no conflicts to declare.

## Supplementary Material

SC-013-D1SC06045G-s001
