# Supplementary material for: A BINOL-phosphoric acid and metalloporphyrin derived chiral covalent organic framework for enantioselective α-benzylation of aldehydes
Source: Chem Sci. 2022 Jan 14;13(7):1906–11. doi: 10.1039/d1sc06045g (PMC8848806; doi:10.1039/d1sc06045g)
Supplement: SC-013-D1SC06045G-s001 [file SC-013-D1SC06045G-s001.pdf]

Electronic Supplementary Material (ESI) for Chemical Science.
This journal is © The Royal Society of Chemistry 2022

Electronic Supplementary Information for

BINOL-phosphoric acid and metalloporphyrin derived chiral covalent organic framework for enantioselective α-benzylation of aldehydes

Hui-Chao Ma, Ya-Nan Sun, Gong-Jun Chen* and Yu-Bin Dong*

College of Chemistry, Chemical Engineering and Materials Science, Collaborative Innovation Center of Functionalized Probes for Chemical Imaging in Universities of Shandong, Key Laboratory of Molecular and Nano Probes, Ministry of Education, Shandong Normal University, Jinan 250014, P. R. China.

Email: gongjchen@126.com, yubindong@sdnu.edu.cn

Contents

1. Instruments and materials (page S2)

2. Synthesis of monomers (page S2)

3. Synthesis of (*R*)- and (*S*)-CuTAPBP-COF (page S4)

4. General procedure for synthesis of (*R*)- and (*S*)-MPP (page S4)

5. Gram-scale preparation (page S5)

6. Figures S1-S12 (page S5)

7. Tables S1-S2 (page S25)

8. References (page S34)

1. Instruments and materials

The reagents and solvents employed were commercially available and used without further purification. 5,10,15,20-tetrakis(4-aminophenyl)porphyrin-Cu-(II) (Cu-TAPP)[1] and (*R*)- and (*S*)-DCDB[2] monomers were prepared according to the reported methods. The powder diffractometer (XRD) patterns were collected by a D8 ADVANCEX-ray with Cu Kα radiation (λ = 1.5405 Å). The total surface areas of the catalysts were measured by the BET (Brunauer–Emmer–Teller) method using N_2 adsorption at 77 K, this was done by the Micromeritics ASAP 2000 sorption/desorption analyzer. Inductively coupled plasma (ICP) measurement was conducted on an IRIS Intrepid (II) XSP and NU AttoM spectrometer. HRTEM (High resolution transmission electron microscopy) analysis was performed on a JEOL 2100 Electron Microscope at an operating voltage of 200 kV. Scanning electron microscopy (SEM) images were taken on a SUB010 scanning electron microscope with acceleration voltage of 20 kV. Elemental analyses for C, H and N were obtained on a Perkin-Elmer analyzer model 240. Infrared (IR) samples were prepared as KBr pellets, and spectra were obtained in the 400-4000 cm^{-1} range using a Perkin-Elmer 1600 FTIR spectrometer. ^{13}C NMR spectra were recorded on a MERCURY plus 400 spectrometer operating at resonance frequencies of 400 MHz. Thermogravimetric analyses (TGA) were carried out under flowing nitrogen at a heating rate of 10 °C·min^{-1} on a TA Instrument Q5 analyzer. The solid-state CD spectra were recorded on a J-815 spectropolarimeter (Jasco, Japan). XPS spectra were obtained from PHI Versaprobe II. UV-vis spectrum was recorded on a Cary 5000 UV-vis spectrophotometer (Varian, USA). CD spectra were recorded on a J-815 spectropolarimeter (Jasco, Japan). Gas chromatography (GC) analysis was performed on an Agilent 7890B GC. Enantiomer ratios were determined by chiral HPLC analysis using a Shimadzu LC-10AT VP series and a Shimadzu LC-10A VP UV-vis. Photothermal performance was evaluated by xenon lamp (300 W with the intensity of 2.5 W cm^{-2}).

2. Synthesis of monomers

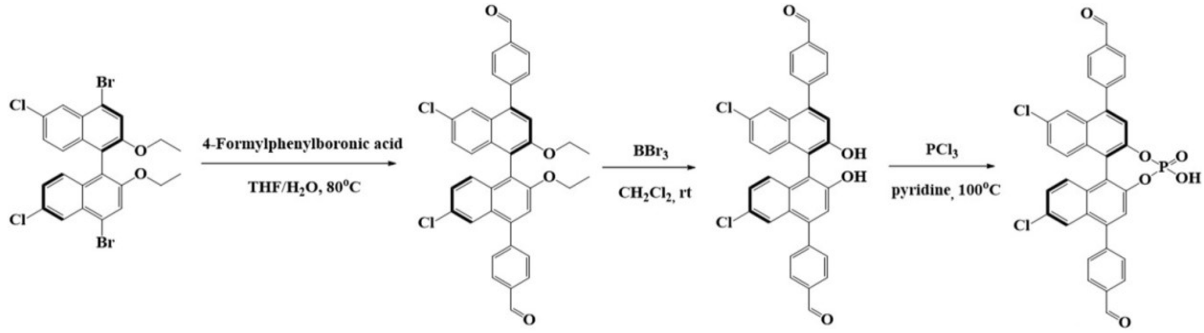

(*R*)-DCDB (*R*)-BINOLDE-DA (*R*)-BINOLDH-DA (*R*)-BINOLPA-DA

Under nitrogen, a mixture of (*R*)-DCDB (0.57 g, 1.0 mmol), 4-formylphenylboronic acid (0.36 g, 2.0 mmol), K_2CO_3 (0.82 g, 6.0 mmol) and $Pd[P(Ph)_3]_4$ (0.08 g, 0.06 mmol) in anhydrous THF (30 mL) and water (10 mL) was heated at 80 °C for 36 h. Then the reaction mixture was cooled to room temperature. After concentration in vacuum, the residue was extracted with DCM. The organic layer was collected, dried over anhydrous $MgSO_4$, and concentrated under reduced pressure. The crude product was purified by column chromatography on silica gel (1:1, DCM/PE, v/v) to yield (*R*)-**BINOLDE-DA** as a light-yellow solid (Yield, 65%). ESI-MS: calculated for $[M+H]^+$: 619.1365, found 619.1046. ^{1}H NMR (400 MHz, $CDCl_3$): δ 10.23 (s, 2H), 8.15 (d, J = 8.2 Hz, 6H), 7.80 (d, J = 8.2 Hz, 4H), 7.53-7.65 (s, 4H), 7.34 (s, 2H), 4.58 (q, 4H), 1.36 (t, J = 7.0 Hz, 6H). ^{13}C NMR (400 MHz, $CDCl_3$): δ 192.02, 156.13, 147.06, 136.76, 135.31, 132.97, 130.85, 129.06, 128.24, 127.75, 126.28, 122.85, 120.63, 118.94, 62.7, 15.2.

To a solution of boron tribromide (6.0 mmol, 570 μL) in DCM (10 mL) was added (*R*)-**BINOLDE-DA** (0.62 mg, 1.0 mmol) in DCM (10 mL). The mixture was stirred at room temperature for 24 h. Quenching reaction with ice water, the organic layer was separated, and the aqueous layer was completely extracted with DCM, dried with $MgSO_4$ and concentrated under reduced pressure to provide (*R*)-**BINOLDH-DA** (Yield, 87%) as a beige crystalline solid. ESI-MS: calculated for $[M+H]^+$: 563.0739, found 563.1012. ^{1}H NMR (400 MHz, $CDCl_3$): δ 10.17 (s, 2H), 8.06 (d, J = 8.2 Hz, 6H), 7.80 (d, J = 8.2 Hz, 4H), 7.42-7.62 (s, 4H), 7.33 (s, 2H), 5.50 (s, 2H). ^{13}C NMR (400 MHz, $CDCl_3$): δ 192.03, 155.21, 146.84, 137.62, 135.35, 132.75, 131.26, 129.55, 128.07, 127.04, 126.76, 124.31, 118.73, 115.60.

To a solution of (*R*)-**BINOLDH-DA** (0.56 mg, 1.0 mmol) in anhydrous pyridine (20 mL) was added $POCl_3$ (457 μL, 4.9 mmol) slowly at 0 °C. After stirring at 100 °C for 48 h, the reaction mixture was quenched by addition of distilled water (4.5 mL) slowly at 0 °C and then stirred at 110 °C for 48 h. After cooling to room temperature, the mixture was acidified to pH = 5 with 6 M HCl and then extracted with DCM. The organic layer was washed with brine, dried over $MgSO_4$, and concentrated in vacuum. The crude product was purified by column chromatography on silica gel (1:1, PE-EA, v/v) to yield (*R*)-**BINOLPA-DA** as a light-yellow solid (Yield, 76%). ESI-MS: calculated for $[M+H]^+$: 625.0296, found 625.0286. ^1H NMR (400 MHz, $CDCl_3$): δ 11.92 (s, 1H), 10.20 (s, 2H), 8.15 (d, J = 8.2 Hz, 6H), 7.82 (d, J = 8.2 Hz, 4H), 7.53-7.65 (s, 4H), 7.32 (s, 2H). ^{13}C NMR (400 MHz, $CDCl_3$): δ 191.88, 148.25, 146.32, 136.98, 135.21, 132.42, 129.00, 128.54, 127.30, 126.77, 124.85, 120.63, 118.94, 117.17, 114.50. (*S*)-**BINOLPA-DA** was synthesized following the same method mentioned above except that (*S*)-DCDB was used instead of (*R*)-DCDB.

3. Synthesis of (*R*)- and (*S*)-CuTAPBP-COF

A mixture of Cu(II)-TAPP (36.6 mg, 0.05 mmol), (*R*)- or (*S*)-**BINOLPA-DA** (61.6 mg, 0.1 mmol) and acetic acid (9 M, 0.2 mL) in ethanol (1.5 mL)/mesitylene (0.3 mL) was heated at 120°C for 3 days in a sealed Schlenk (10 mL) in N_2 to afford corresponding (*R*)- or (*S*)-**CuTAPBP-COF** as the purple black crystalline solids. After stayed in the vacuum chamber (100 °C) for 12 h, the activated samples were obtained (Yield, 58%).

4. General procedure for synthesis of (*R*)- and (*S*)-MPP

A mixture of propanal (79 μL, 0.5 mmol), 4-(bromomethyl)pyridine (86 mg, 0.5 mmol), 2,6-lutidine (88 μL, 0.75 mmol) and CCOF catalyst (10.0 mg, 0.17 mol COF %; 1.8 mol % Cu equiv, 1.7 mol % P equiv) in CH_3OH (1.5 mL) was stirred at room temperature under visible-light irradiation (λ = 420 nm, 300 W xenon lamp with the intensity at 2.5 W cm^{-2}, 30 cm away from the reaction vessel) for 5 h in air to afford the corresponding chiral α-benzyl aldehydes products. Yield was determined by the GC measurement on HP-5 column, and ee was

determined by HPLC with a Chiralcel OD-H column (95 : 5 = *n*-hexane : isopropanol, 1.0 mL min^{-1}, 254 nm), respectively.

5. Gram-scale preparation

A mixture of propanal (18 mmol), 4-(bromomethyl)pyridine (18 mmol), 2,6-lutidine (27 mmol) and CCOF catalyst (360 mg, 0.17 mol COF %; 1.8 mol % Cu equiv., 1.7 mol % P equiv.) in CH_3OH (54 mL) was stirred at room temperature under visible-light irradiation (λ = 420 nm, 300 W xenon lamp with the intensity at 2.5 W cm^{-2}, 30 cm away from the reaction vessel) for 5 h in air to afford the corresponding chiral products. The reaction was monitored by GC analysis.

6. Figures S1-S12

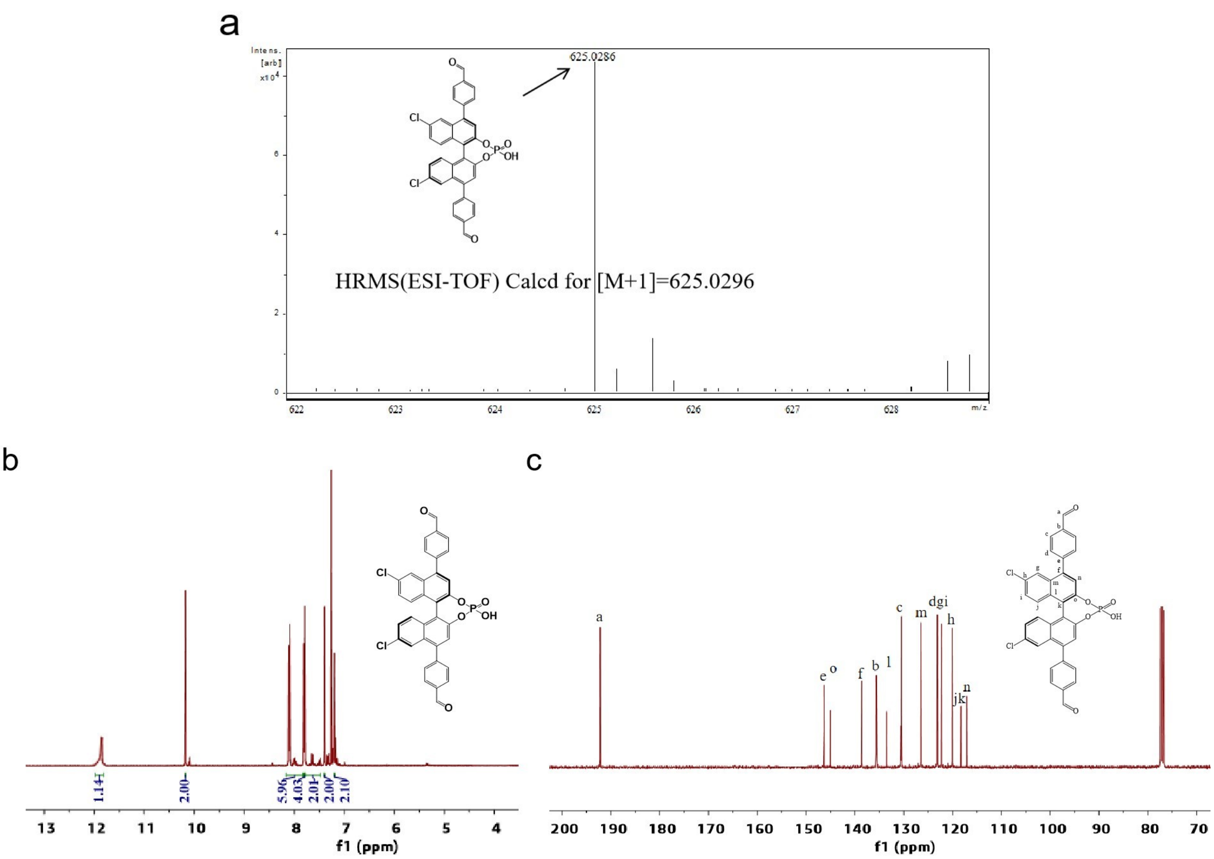

Fig. S1 Characterization of (*R*)-**BINOLPA-DA**. (a) MS spectrum of (*R*)-**BINOLPA-DA**. (b) ^{1}H NMR spectrum of (*R*)-**BINOLPA-DA**. (c) ^{13}C NMR spectrum of (*R*)-**BINOLPA-DA**.

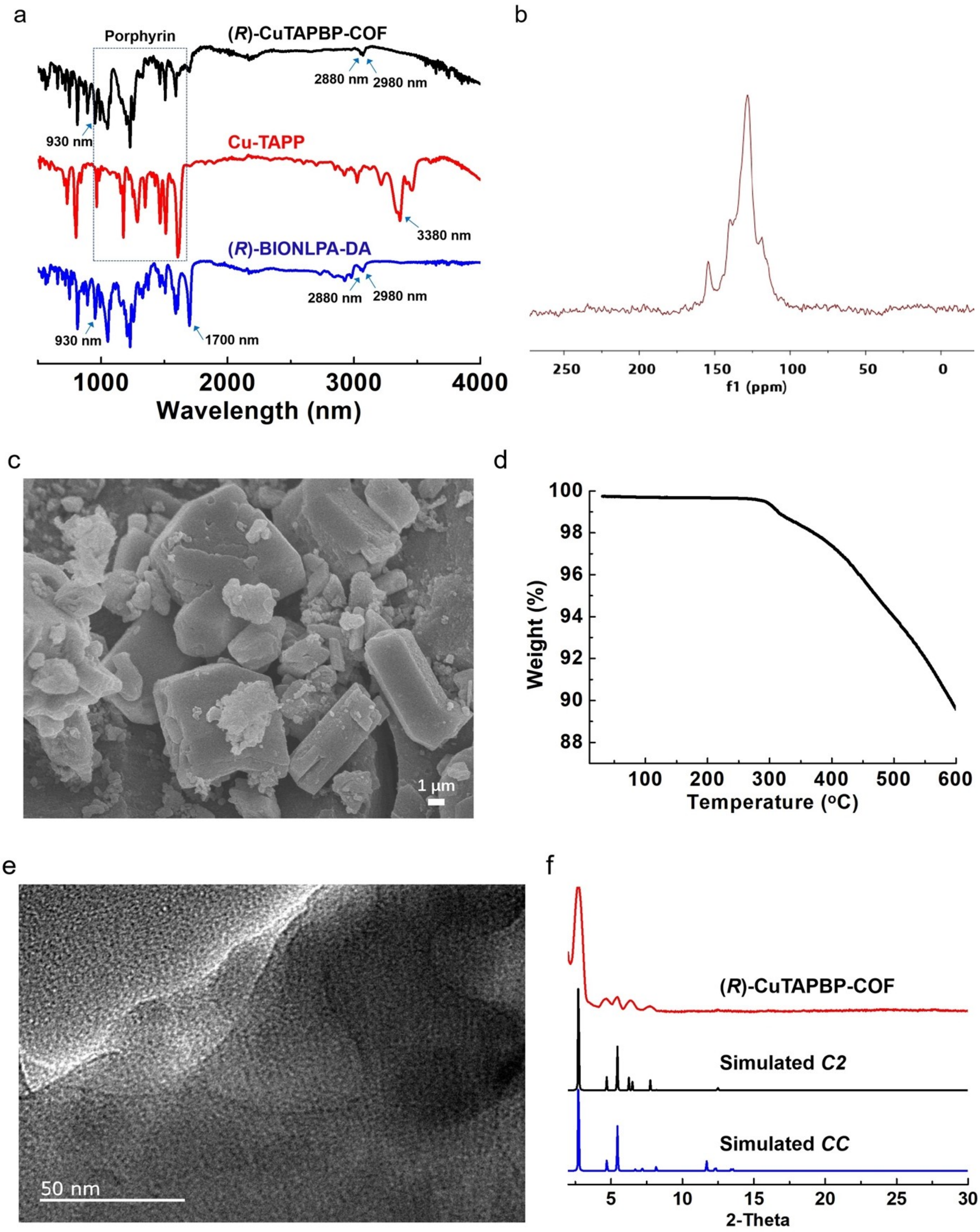

Fig. S2 Characterization of (*R*)-**CuTAPBP-COF**. (a) IR spectra of (*R*)-**CuTAPBP-COF** and its monomers. The IR spectrum of (*R*)-**CuTAPBP-COF** showed that the characteristic C=O stretching vibration of the aldehydes at 1700 cm^{-1} and NH_2 stretching vibration of Cu-TAPP at 3380 cm^{-1} disappeared after the reaction. Meanwhile the P=O stretching vibrations at 930 cm^{-1} and characteristic porphyrin stretching vibrations (1340, 1390, 1490 and 1590

cm^{-1} for porphyrin C=N bonds and 1000 cm^{-1} for porphyrin skeleton vibration) indicated that both the phosphate and porphyrin units exist in CCOF. Elemental Analysis (%) calcd for $C_{112}H_{62}N_8P_2Cu$: C, 85.06; N, 7.09; H, 7.85; found (%): C, 85.04; N, 6.96; H, 7.95. The P content is 1.02 wt% (calcd, 1.06 wt%) and Cu content is 1.11 wt% (calcd, 1.09 wt%) as determined by ICP-AES. (b) ^{13}C CP-MAS NMR spectrum of (*R*)-**CuTAPBP-COF**. The characteristic resonances in a range of 150-115 ppm are associated with the C=N and BINOL units in CCOF; the signals at 160 ppm are assigned to porphyrin unit. (c) SEM image of (*R*)-**CuTAPBP-COF**. (d) TGA trace of (*R*)-**CuTAPBP-COF**. (e) HRTEM image of (*R*)-**CuTAPBP-COF**. (f) Measured and simulated PXRD patterns for **(*R*)-CuTAPBP-COF**. Compared to the pattern generated from the *Cc* space group (blue line), (*R*)-**CuTAPBP-COF** unequivocally crystallizes in the *C*$_2$ space group.

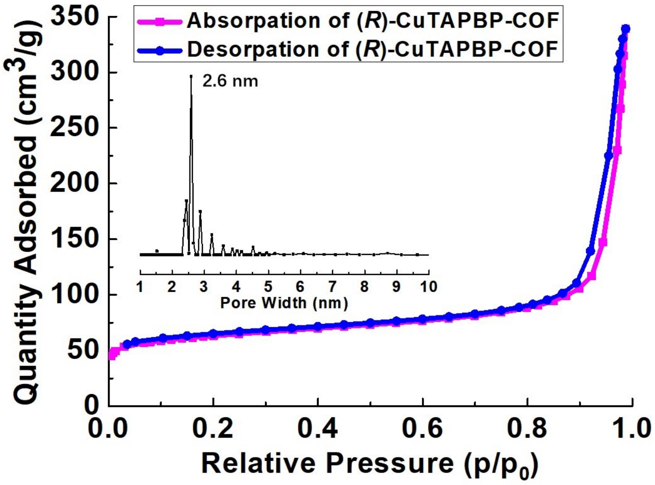

Fig. S3 N_2 adsorption isotherm of (*R*)-**CuTAPBP-COF** at 77 K. Its pore width distribution is inserted.

a

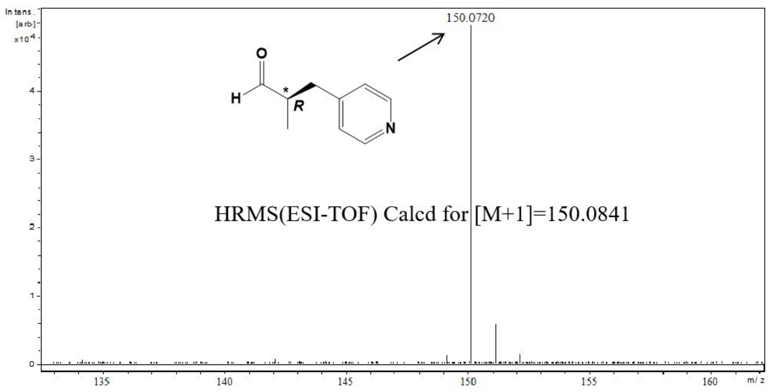

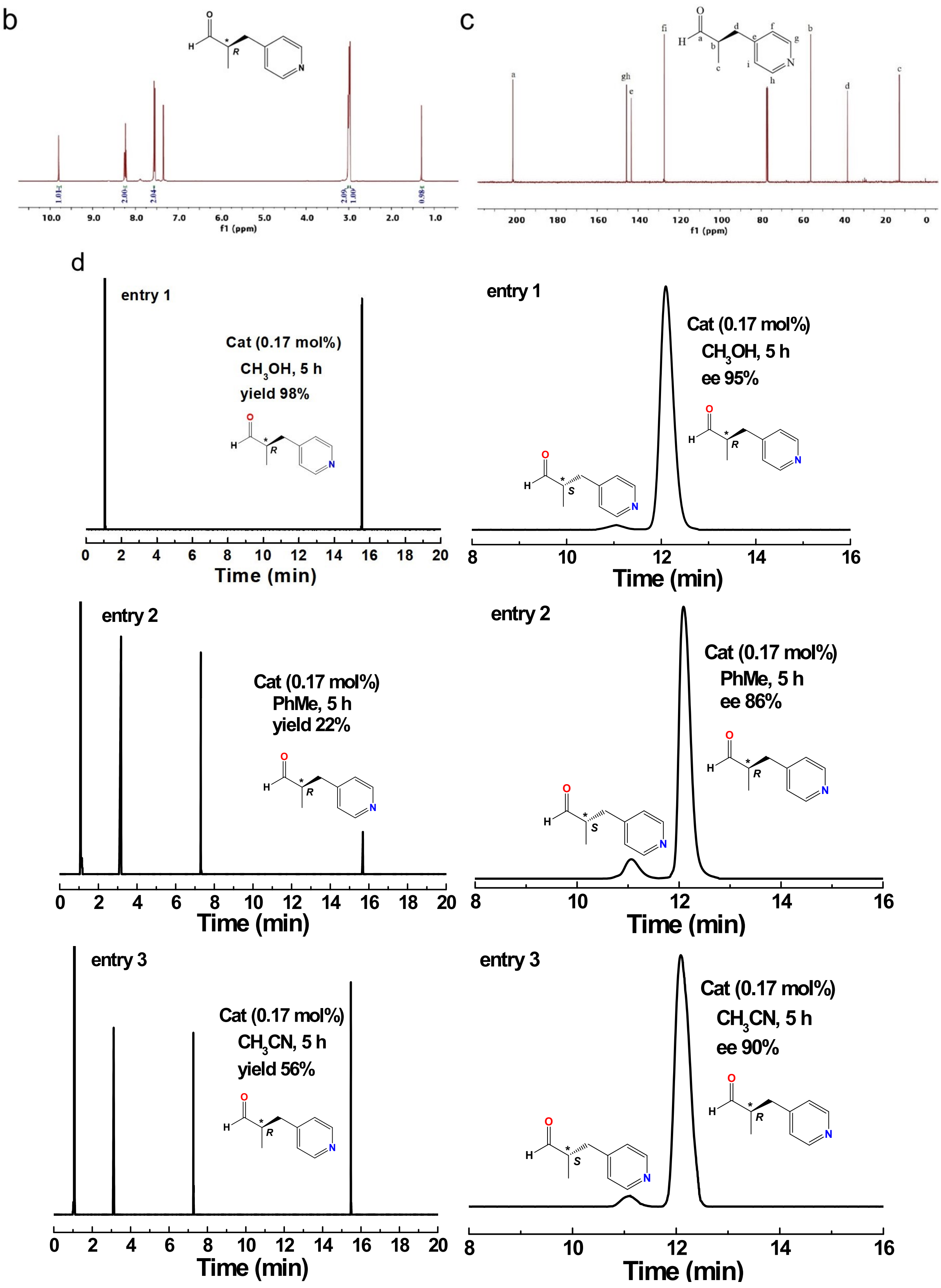

b
c
d
f1 (ppm)
entry 1
Cat (0.17 mol%)
CH3OH, 5 h
yield 98%
ee 95%
entry 2
Cat (0.17 mol%)
PhMe, 5 h
yield 22%
ee 86%
entry 3
Cat (0.17 mol%)
CH3CN, 5 h
yield 56%
ee 90%
Time (min)

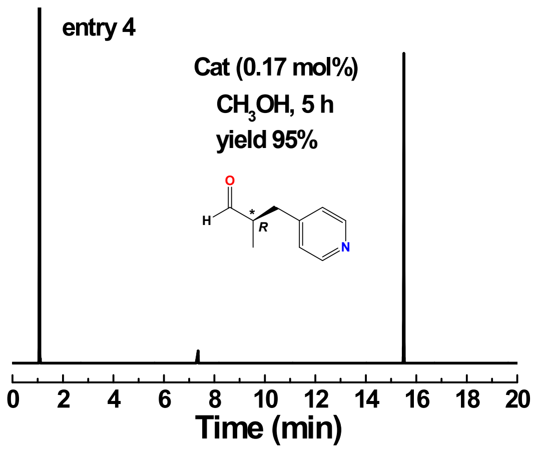
entry 4
Cat (0.17 mol%)
CH3OH, 5 h
yield 95%
O
H
*
R
N
0 2 4 6 8 10 12 14 16 18 20
Time (min)

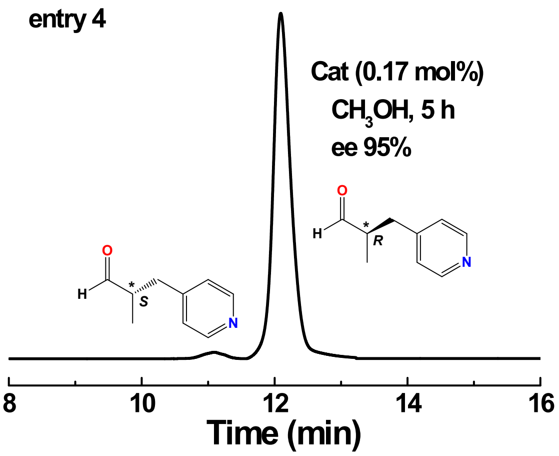
entry 4
Cat (0.17 mol%)
CH3OH, 5 h
ee 95%
O
H
*
R
N
O
H
*
S
N
8 10 12 14 16
Time (min)

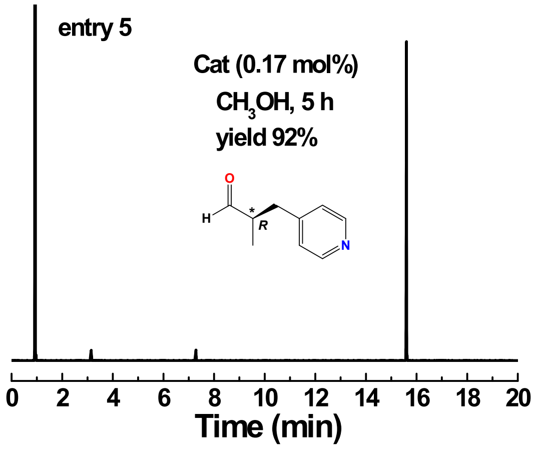
entry 5
Cat (0.17 mol%)
CH3OH, 5 h
yield 92%
O
H
*
R
N
0 2 4 6 8 10 12 14 16 18 20
Time (min)

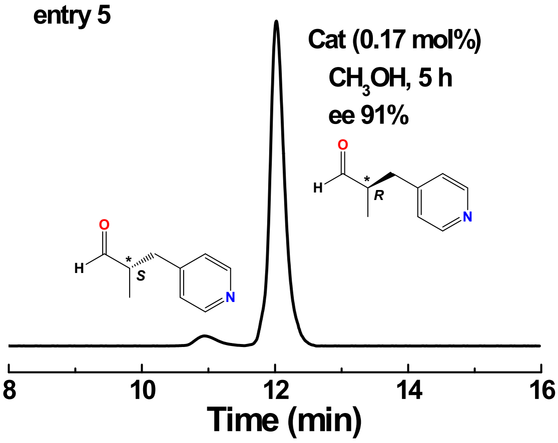
entry 5
Cat (0.17 mol%)
CH3OH, 5 h
ee 91%
O
H
*
R
N
O
H
*
S
N
8 10 12 14 16
Time (min)

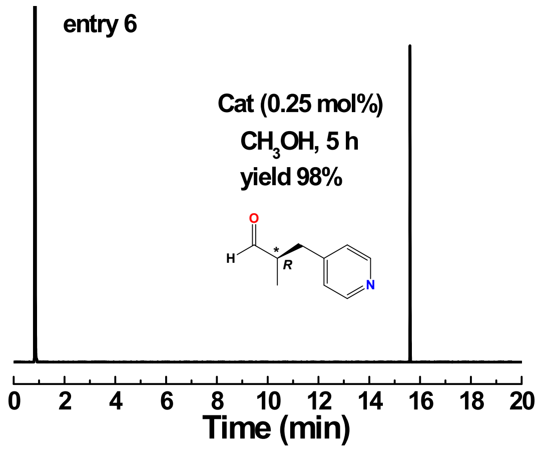
entry 6
Cat (0.25 mol%)
CH3OH, 5 h
yield 98%
O
H
*
R
N
0 2 4 6 8 10 12 14 16 18 20
Time (min)

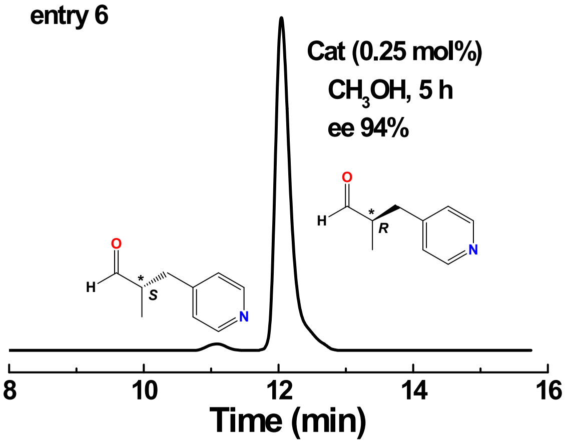
entry 6
Cat (0.25 mol%)
CH3OH, 5 h
ee 94%
O
H
*
R
N
O
H
*
S
N
8 10 12 14 16
Time (min)

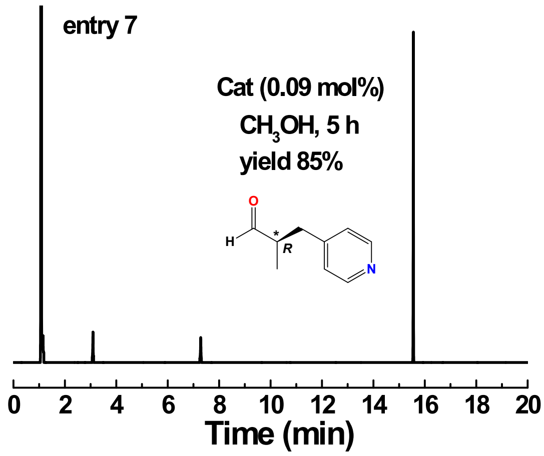
entry 7
Cat (0.09 mol%)
CH3OH, 5 h
yield 85%
O
H
*
R
N
0 2 4 6 8 10 12 14 16 18 20
Time (min)

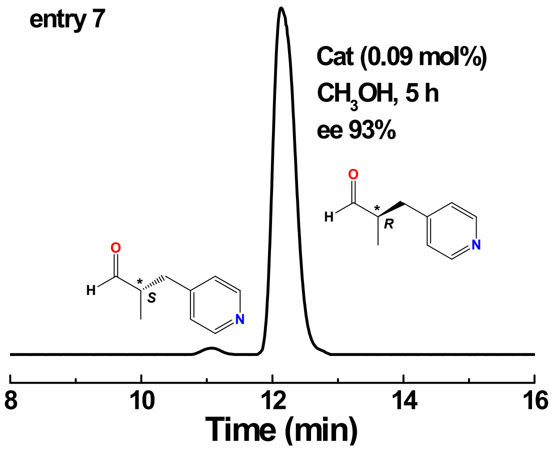
entry 7
Cat (0.09 mol%)
CH3OH, 5 h
ee 93%
O
H
*
R
N
O
H
*
S
N
8 10 12 14 16
Time (min)

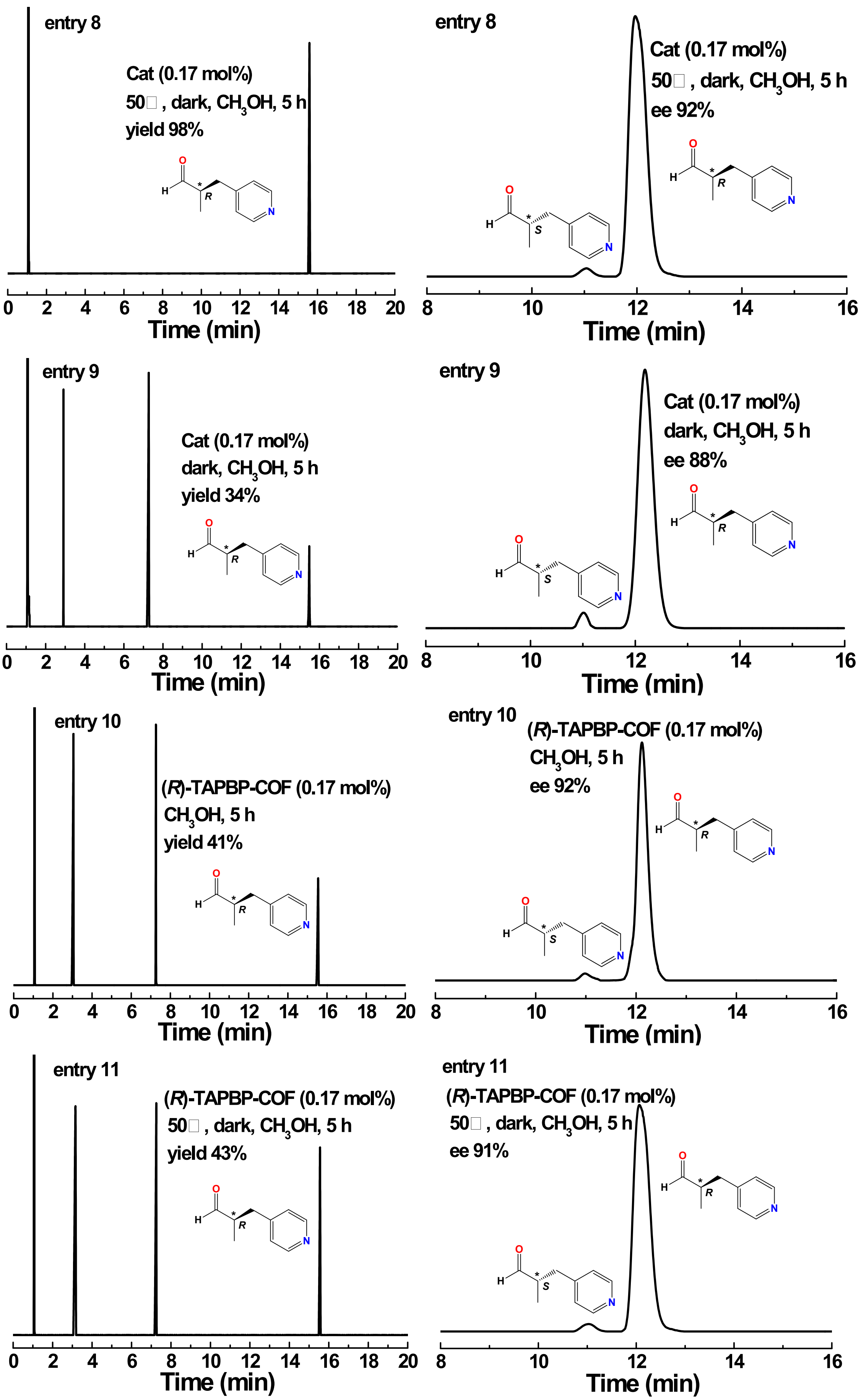
entry 8
Cat (0.17 mol%)
50□ , dark, CH3OH, 5 h
yield 98%
Time (min)
entry 8
Cat (0.17 mol%)
50□ , dark, CH3OH, 5 h
ee 92%
Time (min)
entry 9
Cat (0.17 mol%)
dark, CH3OH, 5 h
yield 34%
Time (min)
entry 9
Cat (0.17 mol%)
dark, CH3OH, 5 h
ee 88%
Time (min)
entry 10
(R)-TAPBP-COF (0.17 mol%)
CH3OH, 5 h
yield 41%
Time (min)
entry 10
(R)-TAPBP-COF (0.17 mol%)
CH3OH, 5 h
ee 92%
Time (min)
entry 11
(R)-TAPBP-COF (0.17 mol%)
50□ , dark, CH3OH, 5 h
yield 43%
Time (min)
entry 11
(R)-TAPBP-COF (0.17 mol%)
50□ , dark, CH3OH, 5 h
ee 91%
Time (min)

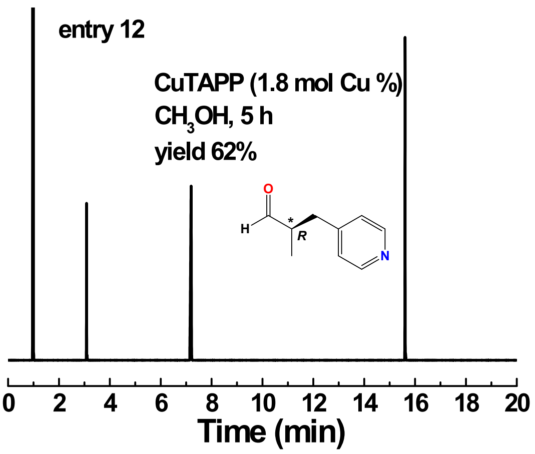
entry 12
CuTAPP (1.8 mol Cu %)
CH_3OH, 5 h
yield 62%
0 2 4 6 8 10 12 14 16 18 20
Time (min)

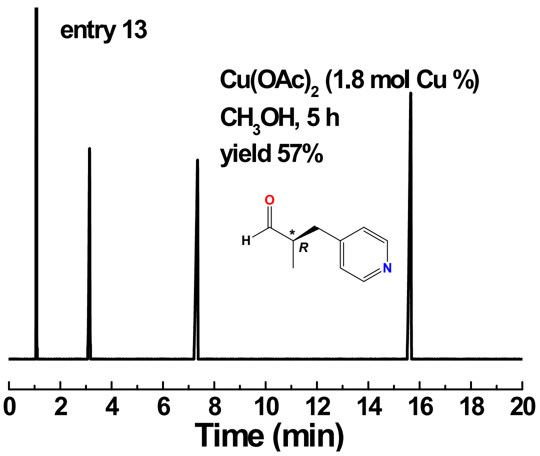
entry 13
$Cu(OAc)_2$ (1.8 mol Cu %)
CH_3OH, 5 h
yield 57%
0 2 4 6 8 10 12 14 16 18 20
Time (min)

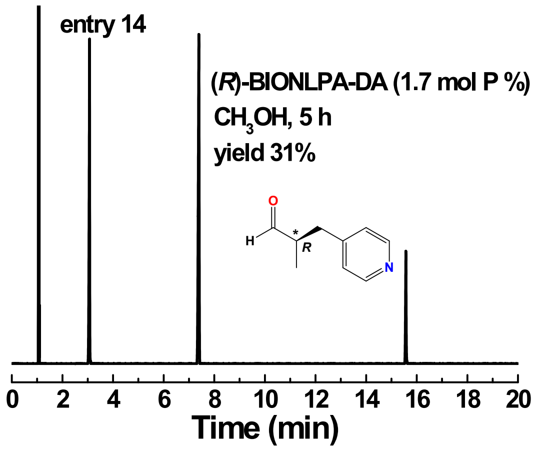
entry 14
(*R*)-BIONLPA-DA (1.7 mol P %)
CH_3OH, 5 h
yield 31%
0 2 4 6 8 10 12 14 16 18 20
Time (min)

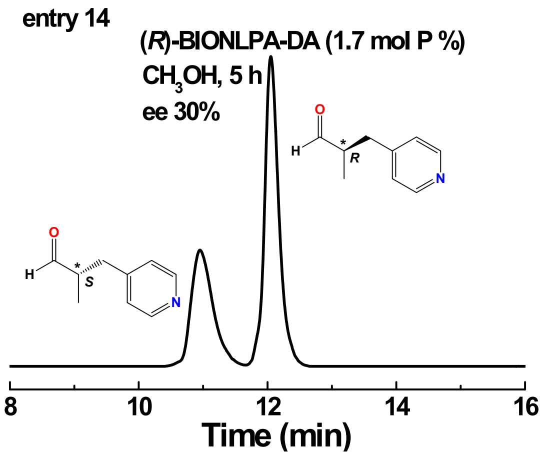
entry 14
(*R*)-BIONLPA-DA (1.7 mol P %)
CH_3OH, 5 h
ee 30%
8 10 12 14 16
Time (min)

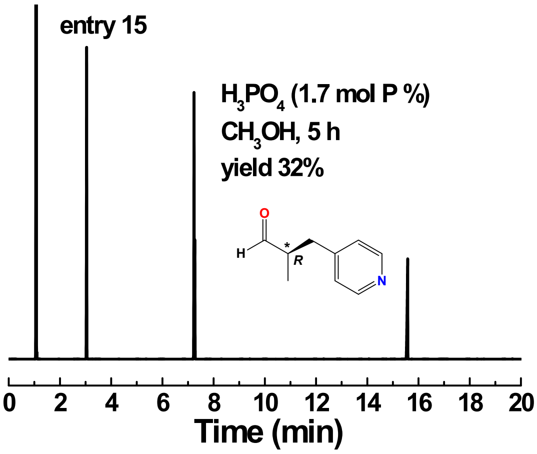
entry 15
H_3PO_4 (1.7 mol P %)
CH_3OH, 5 h
yield 32%
0 2 4 6 8 10 12 14 16 18 20
Time (min)

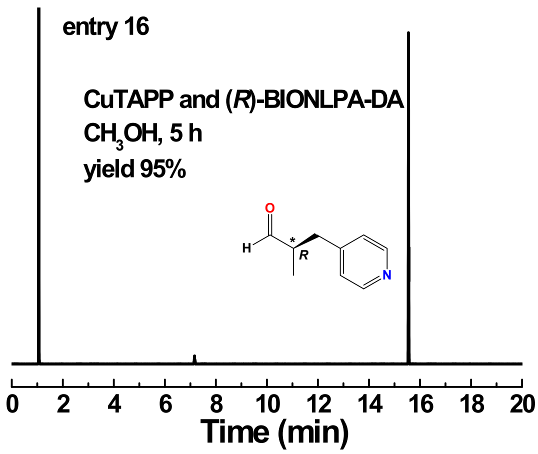
entry 16
CuTAPP and (*R*)-BIONLPA-DA
CH_3OH, 5 h
yield 95%
0 2 4 6 8 10 12 14 16 18 20
Time (min)

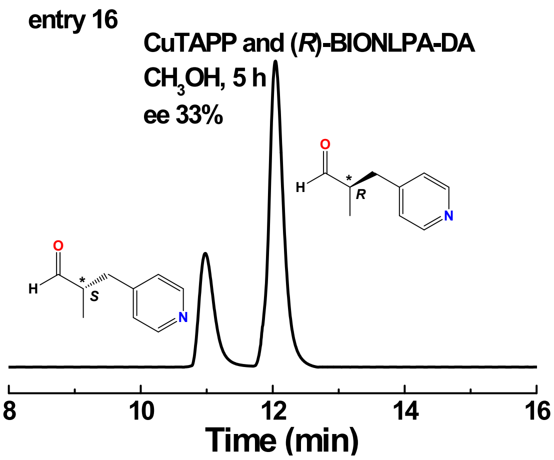
entry 16
CuTAPP and (*R*)-BIONLPA-DA
CH_3OH, 5 h
ee 33%
8 10 12 14 16
Time (min)

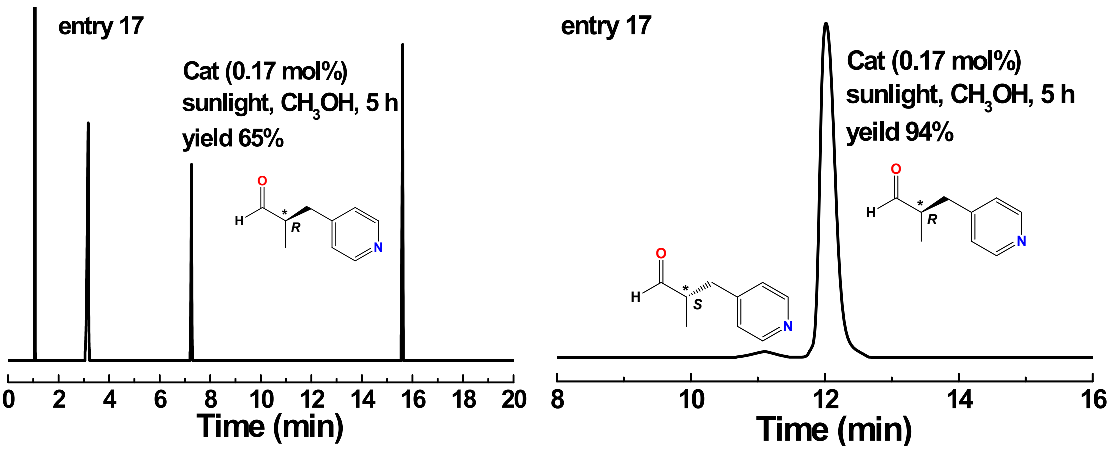

Fig. S4 Characterization of (*R*)-**MPP** (for Table 1). (a) MS spectrum of (*R*)-**MPP**. (b) ^{1}H NMR spectrum of (*R*)-**MPP**. (c) ^{13}C NMR spectrum of (*R*)-**MPP**. (d) Yields and ee values of the asymmetric *α*-benzylation of propanal with 4-(bromomethyl)pyridine catalyzed by (*R*)-**CuTAPBP-COF** (for Table 1). Yield was determined by the GC measurement on HP-5 column, and ee was determined by HPLC with a Chiralcel OD-H column (95 : 5 = *n*-hexane : isopropanol, 1.0 mL min^{-1}, 254 nm), respectively.

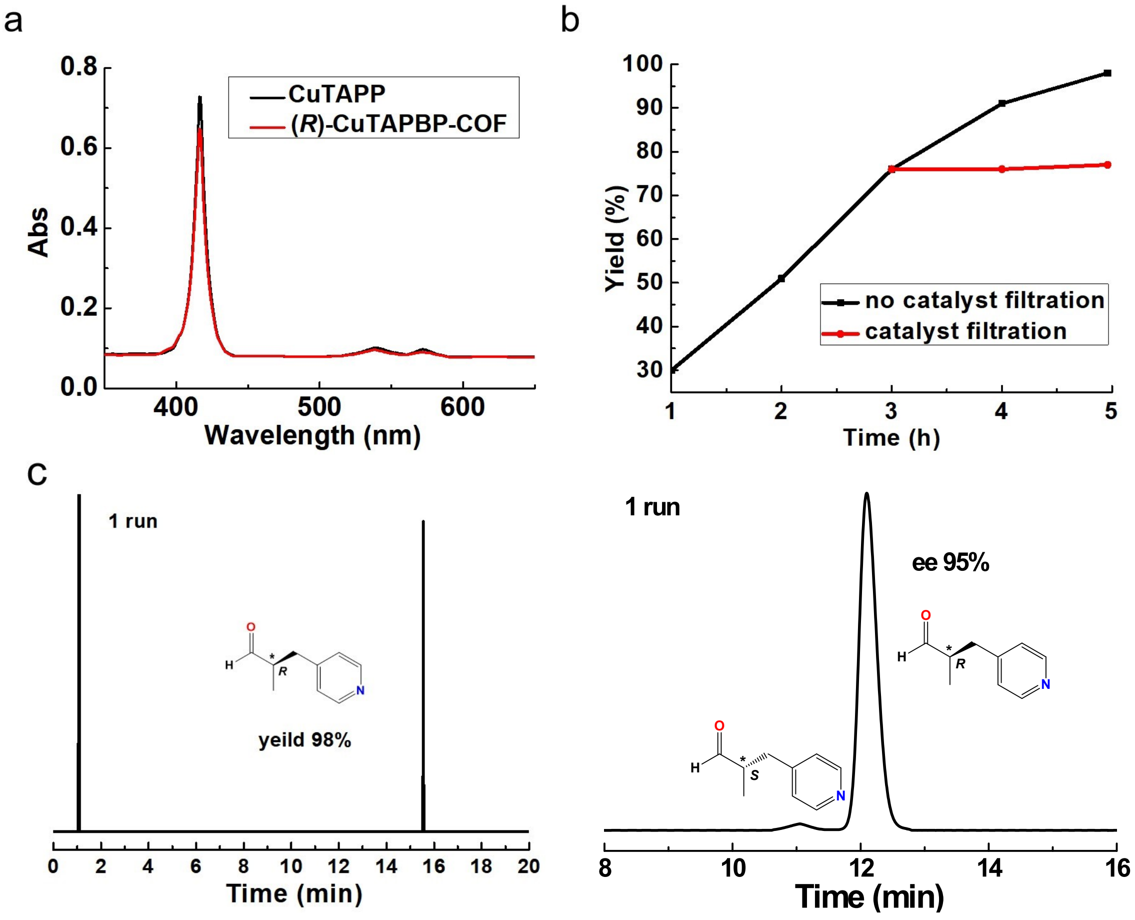

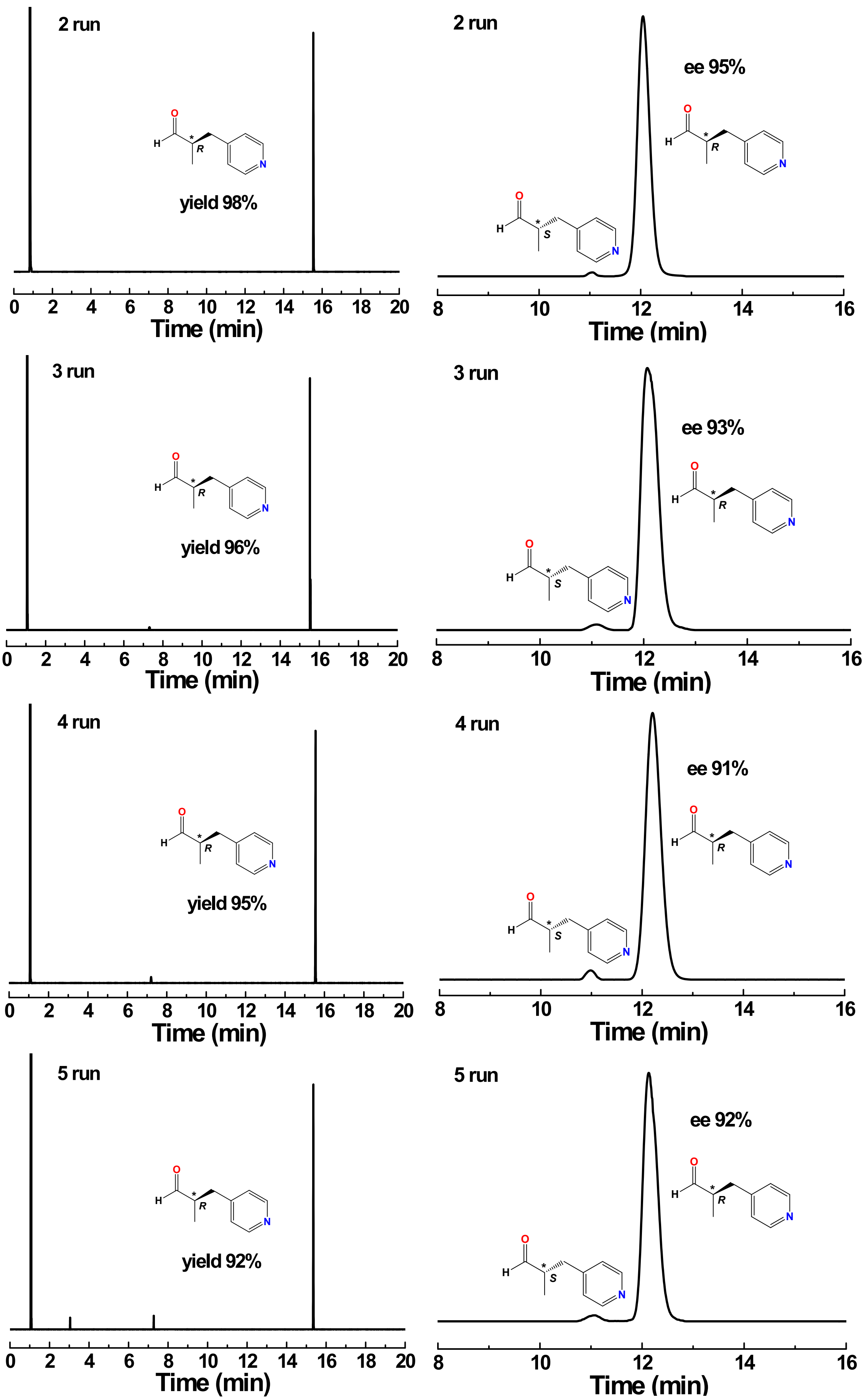
2 run
yield 98%
Time (min)
2 run
ee 95%
Time (min)
3 run
yield 96%
Time (min)
3 run
ee 93%
Time (min)
4 run
yield 95%
Time (min)
4 run
ee 91%
Time (min)
5 run
yield 92%
Time (min)
5 run
ee 92%
Time (min)

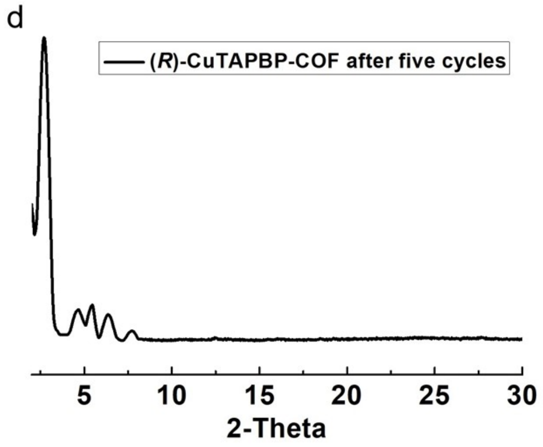

Fig. S5 (a) UV-vis spectra of (*R*)-**CuTAPBP-COF** and Cu-TAPP monomer. (b) Reaction time examination (black line) and leaching test (red line). The solid catalyst was filtrated from the reaction solution after 3 h, whereas the filtrate was transferred to a new vial and the reaction was carried out under the same conditions for an additional 2 h. (c) Catalytic cycles for the (*R*)-**MPP** synthesis by asymmetric *α*-benzylation reaction. Yield was determined by the GC measurement on HP-5 column, and ee was determined by HPLC with a Chiralcel OD-H column (95 : 5 = *n*-hexane : isopropanol, 1.0 mL min^{-1}, 254 nm). (d) PXRD pattern of the (*R*)-**CuTAPBP-COF** after five catalytic cycles. After each run, the solid catalyst was readily recovered by centrifugation, washed with ethanol and dichloromethane, and then dried at 90 °C in vacuum for the next catalytic run under the same reaction conditions.

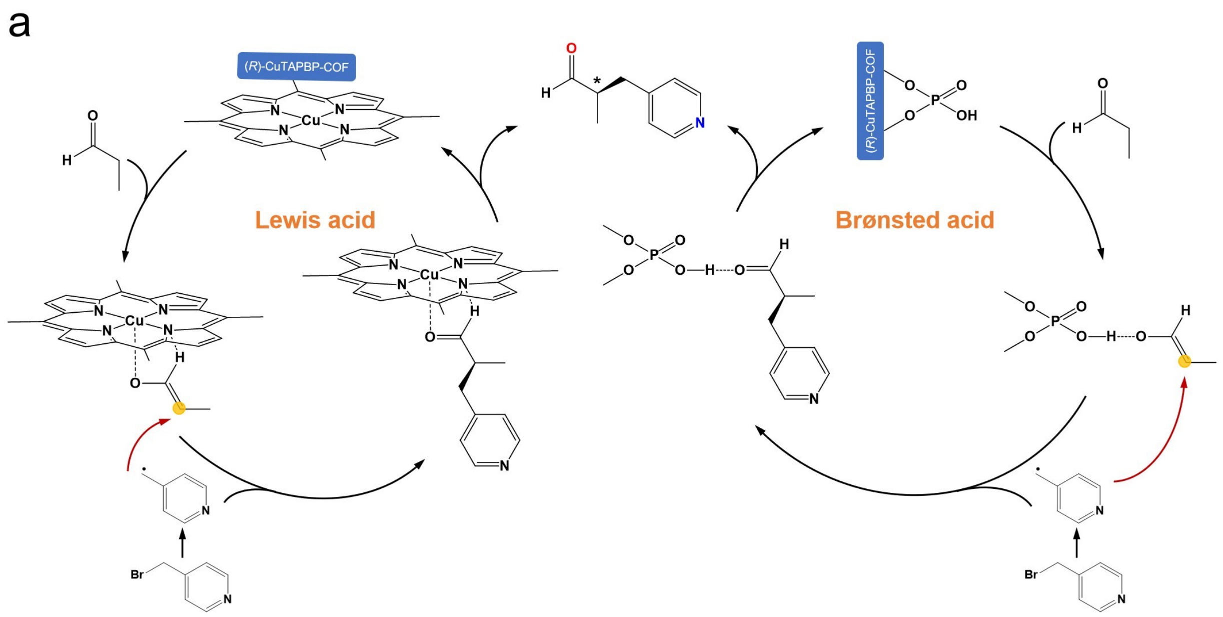

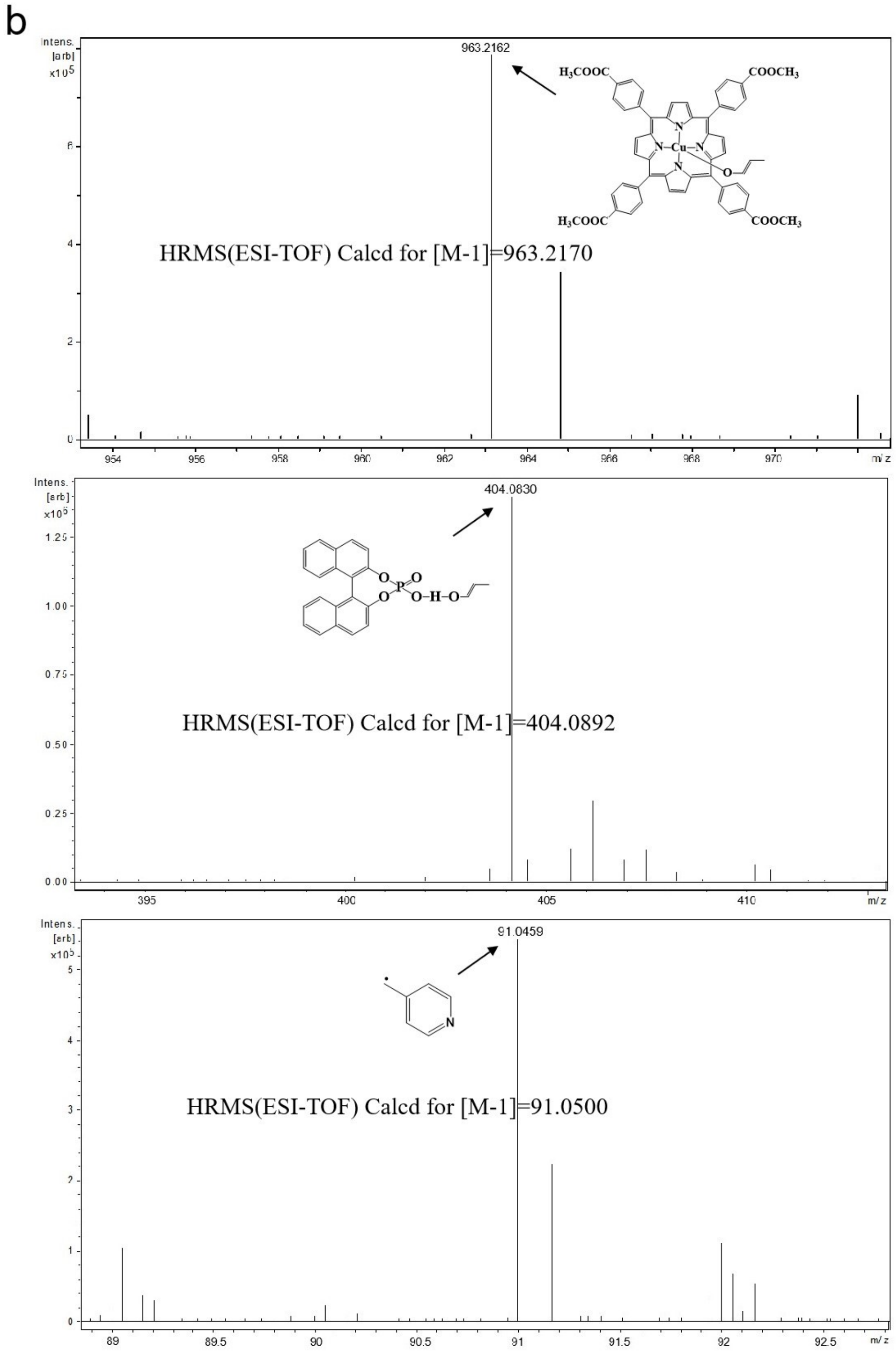

Fig. S6 (a) Proposed mechanism for the (*R*)-**CuTAPBP-COF**-catalyzed α-benzylation of aldehyde based on the model reaction.[3] (b) MS spectra for the corresponding intermediates.

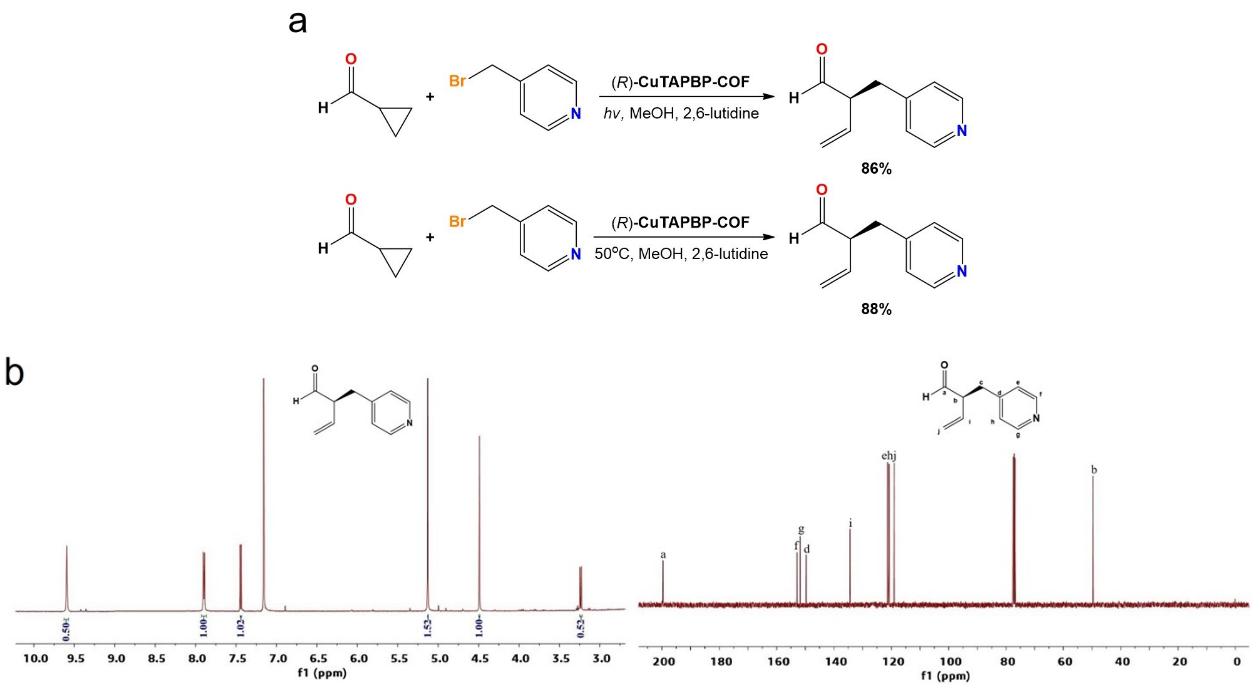

Fig. S7 (a) Radical clock experiment. Light irradiation condition: (*R*)-**CuTAPBP-COF** (10 mg, 0.17 mol%), cyclopropanecarboxaldehyde (37 μL, 0.5 mmol), 4-(bromomethyl)pyridine (86 mg, 0.5 mmol), 2,6-lutidine (88 μL, 0.75 mmol), CH_3OH (1.5 mL), 300 W xenon with a power density of 2.5 W cm^{-2} (λ = 420 nm), 5 h, in air. Yield, 86%. Heating condition: (*R*)-**CuTAPBP-COF** (10 mg, 0.17 mol%), cyclopropanecarboxaldehyde (37 μL, 0.5 mmol), 4-(bromomethyl)pyridine (86 mg, 0.5 mmol), 2,6-lutidine (88 μL, 0.75 mmol), CH_3OH (1.5 mL), 50°C in dark for 5 h in air. Yield, 88%. (b) ^{1}H NMR (left) and ^{13}C NMR (right) spectra of the ring-open product.

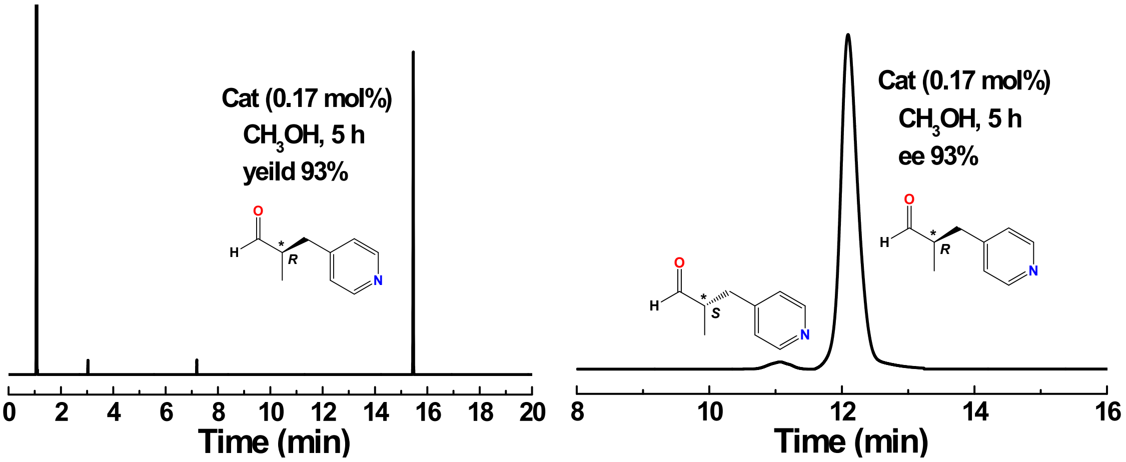

Fig. S8 (*R*)-**MPP** obtained from (*R*)-**CuTAPBP-COF**-catalyzed gram-scale synthesis.

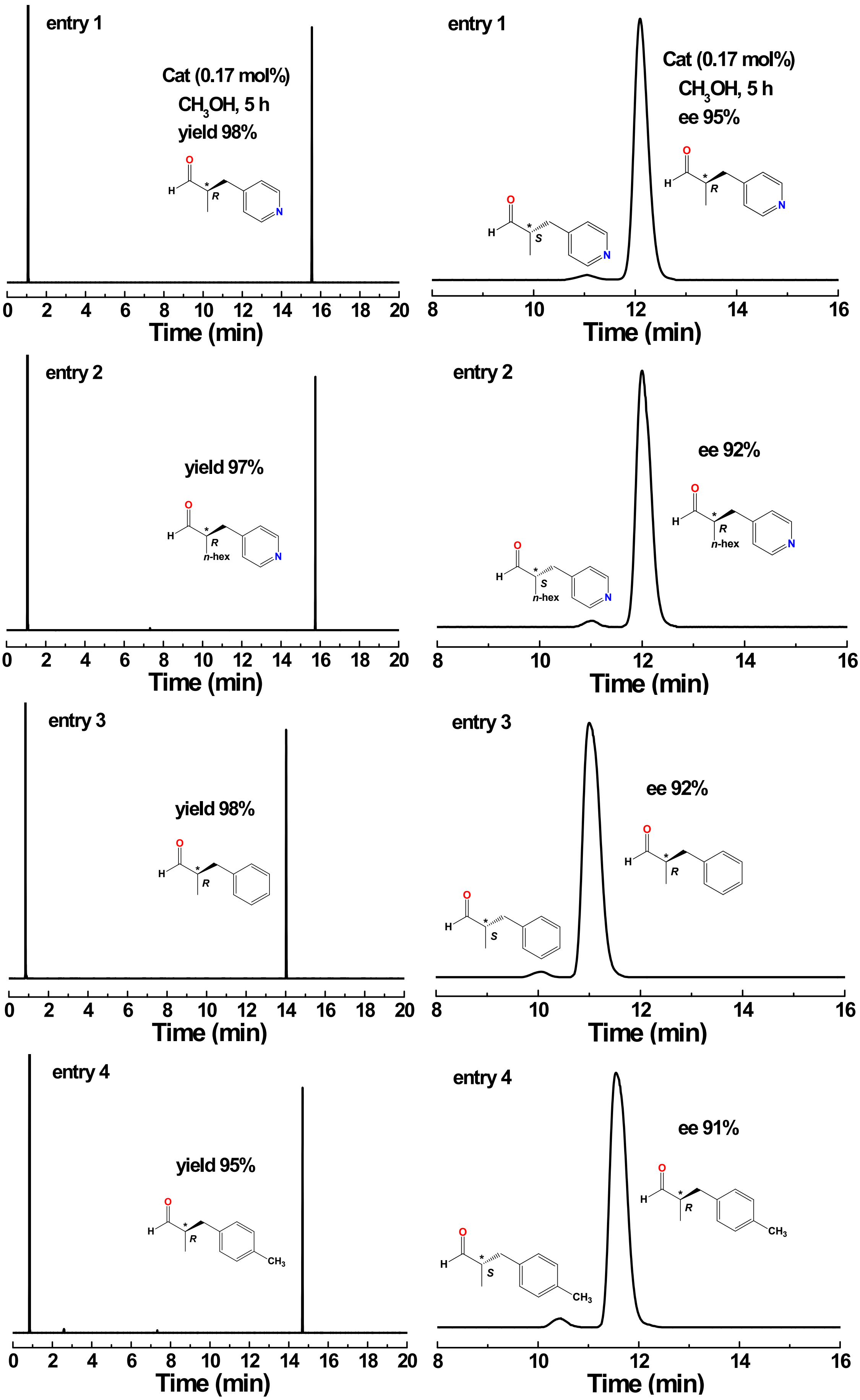
entry 1
Cat (0.17 mol%)
CH_3OH, 5 h
yield 98%
Time (min)
entry 1
Cat (0.17 mol%)
CH_3OH, 5 h
ee 95%
Time (min)
entry 2
yield 97%
n-hex
Time (min)
entry 2
ee 92%
n-hex
Time (min)
entry 3
yield 98%
Time (min)
entry 3
ee 92%
Time (min)
entry 4
yield 95%
CH_3
Time (min)
entry 4
ee 91%
CH_3
Time (min)

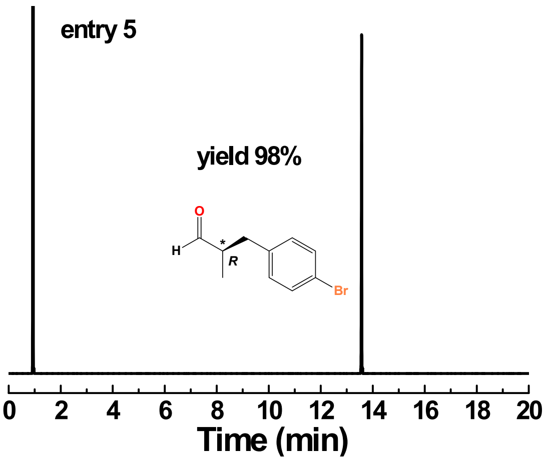
entry 5
yield 98%
0 2 4 6 8 10 12 14 16 18 20
Time (min)

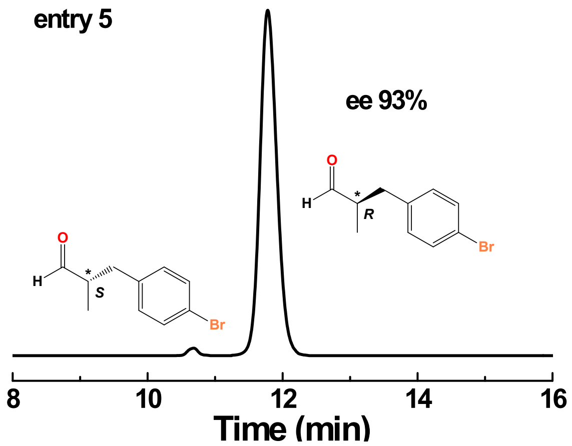
entry 5
ee 93%
8 10 12 14 16
Time (min)

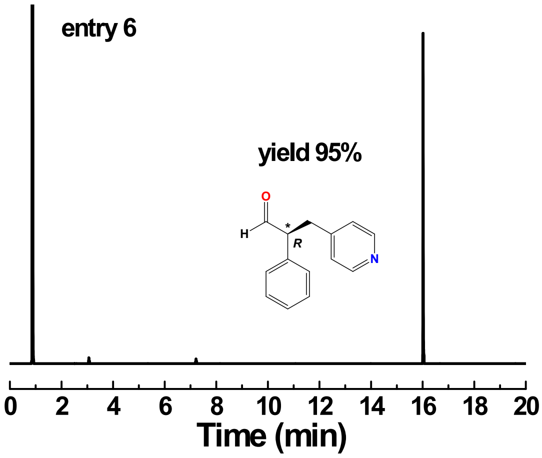
entry 6
yield 95%
0 2 4 6 8 10 12 14 16 18 20
Time (min)

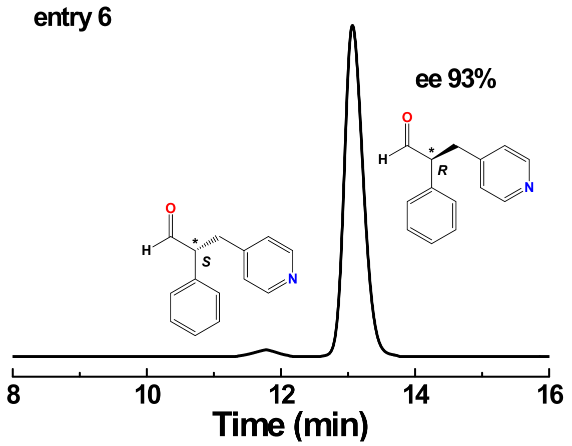
entry 6
ee 93%
8 10 12 14 16
Time (min)

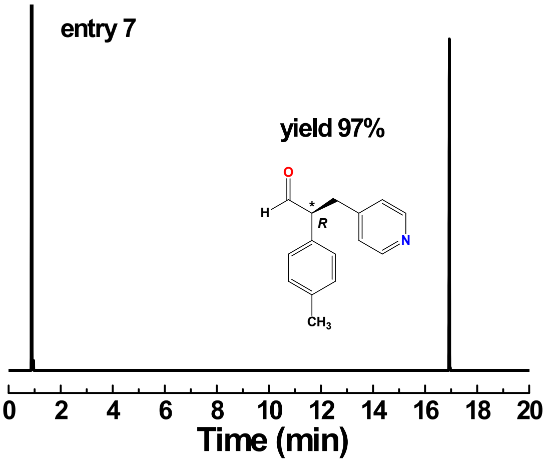
entry 7
yield 97%
0 2 4 6 8 10 12 14 16 18 20
Time (min)

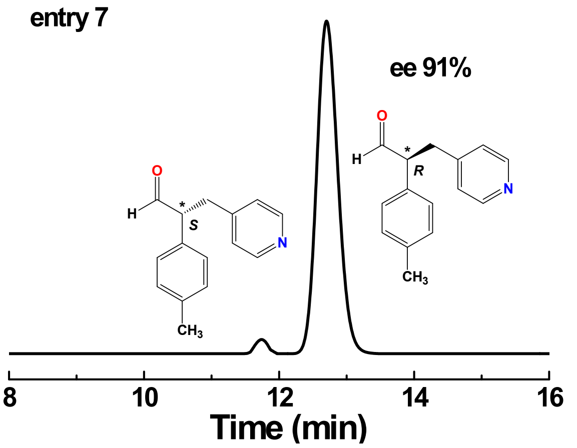
entry 7
ee 91%
8 10 12 14 16
Time (min)

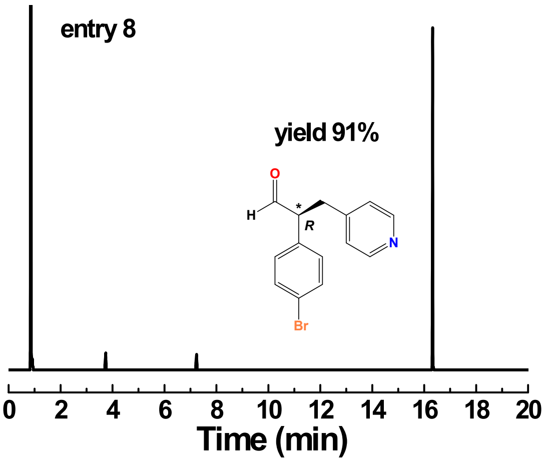
entry 8
yield 91%
0 2 4 6 8 10 12 14 16 18 20
Time (min)

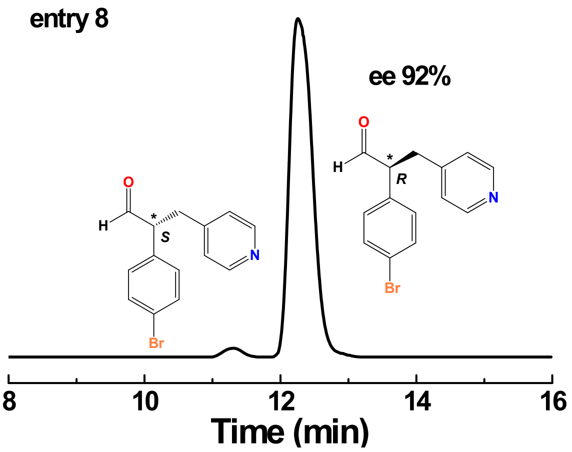
entry 8
ee 92%
8 10 12 14 16
Time (min)

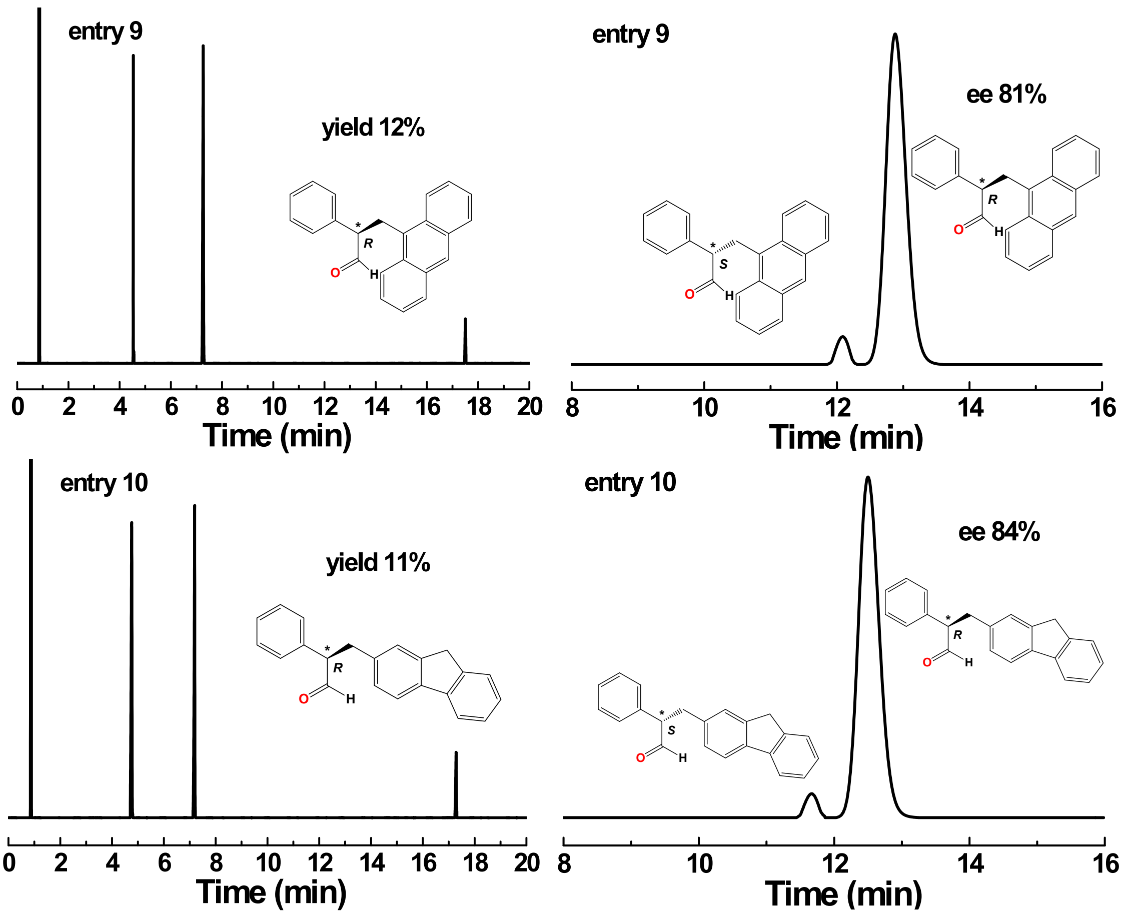

Fig. S9 GC (left) and HPLC (right) results for the expanded *α*-benzylation reactions catalyzed by (*R*)-**CuTAPBP-COF** (For Table 2).

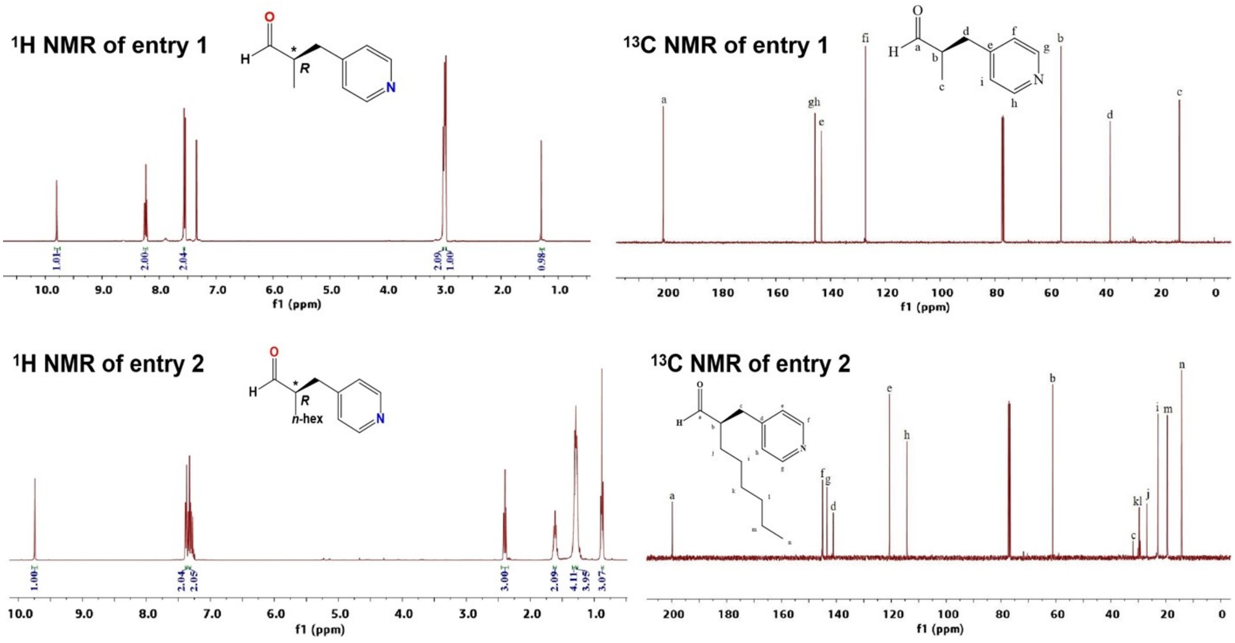

^{1}H NMR of entry 3

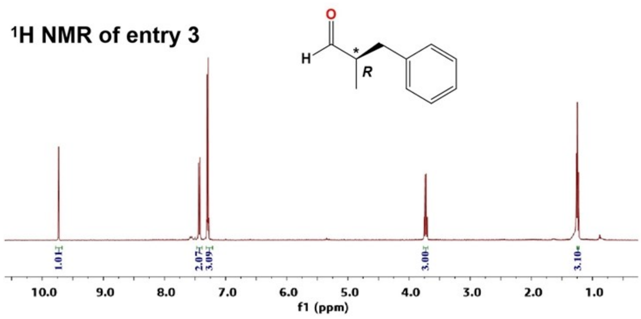

^{13}C NMR of entry 3

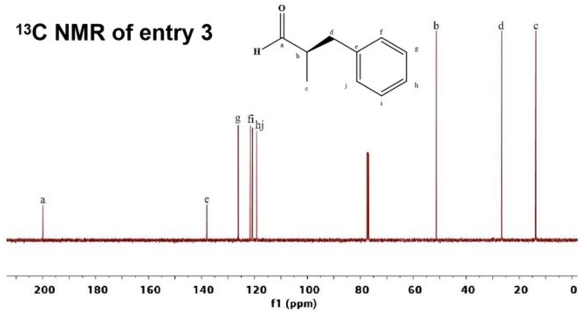

^{1}H NMR of entry 4

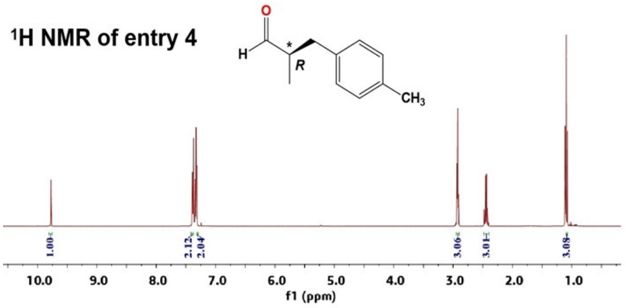

^{13}C NMR of entry 4

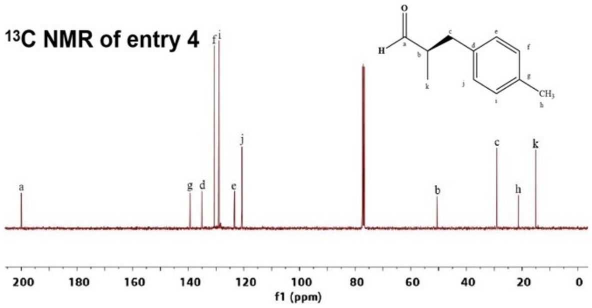

^{1}H NMR of entry 5

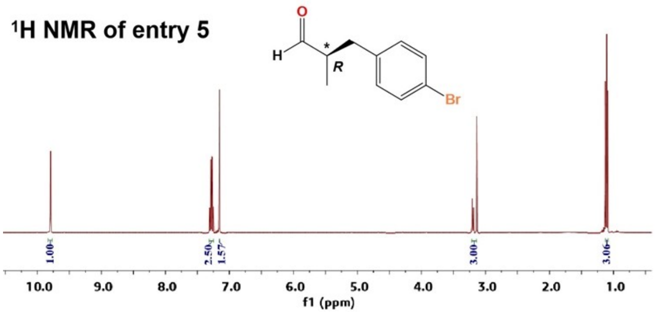

^{13}C NMR of entry 5

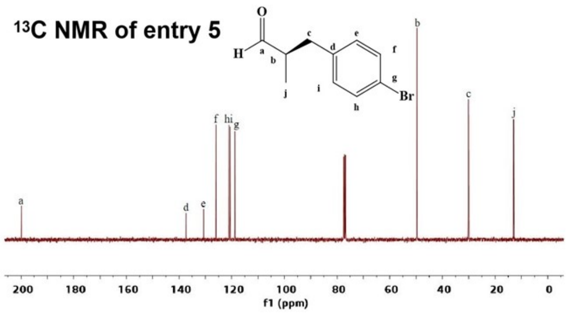

^{1}H NMR of entry 6

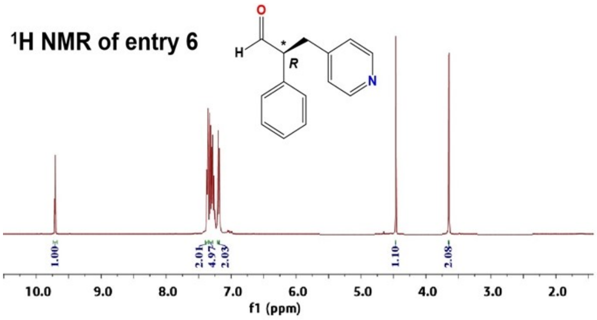

^{13}C NMR of entry 6

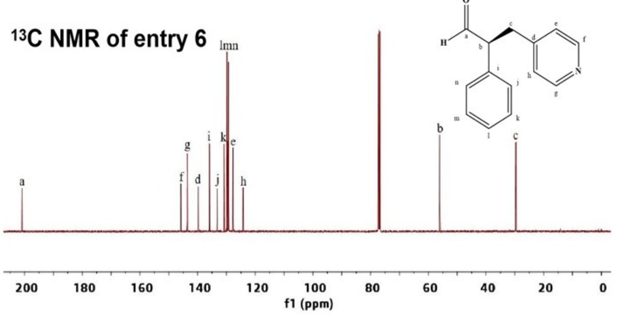

^{1}H NMR of entry 7

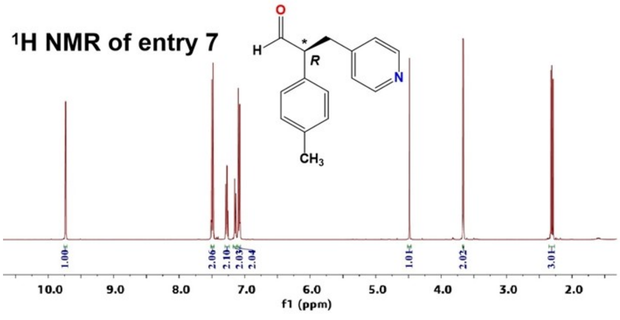

^{13}C NMR of entry 7

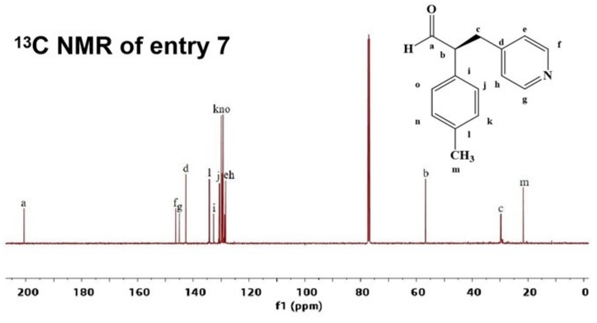

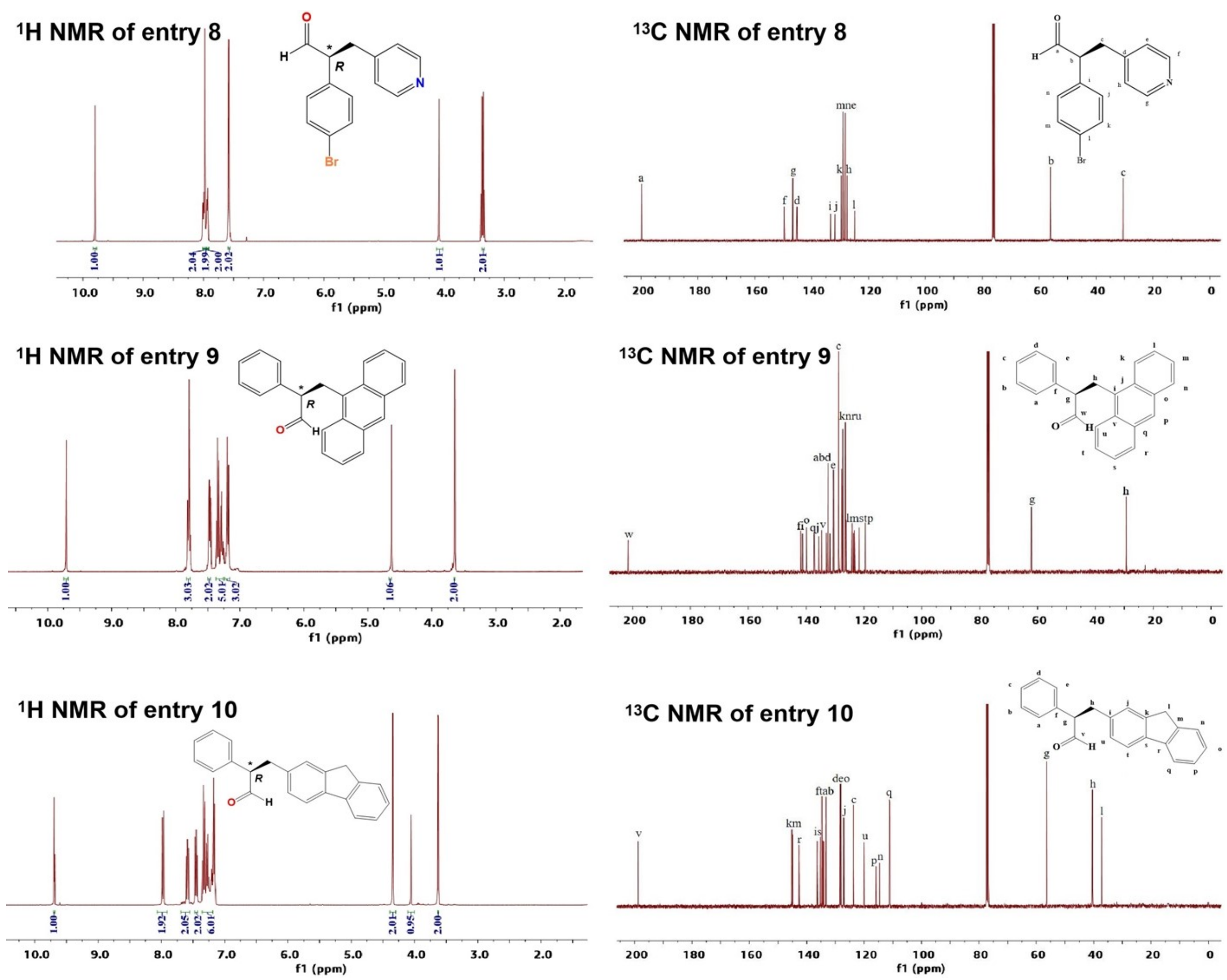

Fig. S10 ^{1}H NMR (left) and ^{13}C NMR (right) spectra for the products obtained from the expanded *α*-benzylation reactions catalyzed by (*R*)-**CuTAPBP-COF** (For Table 2).

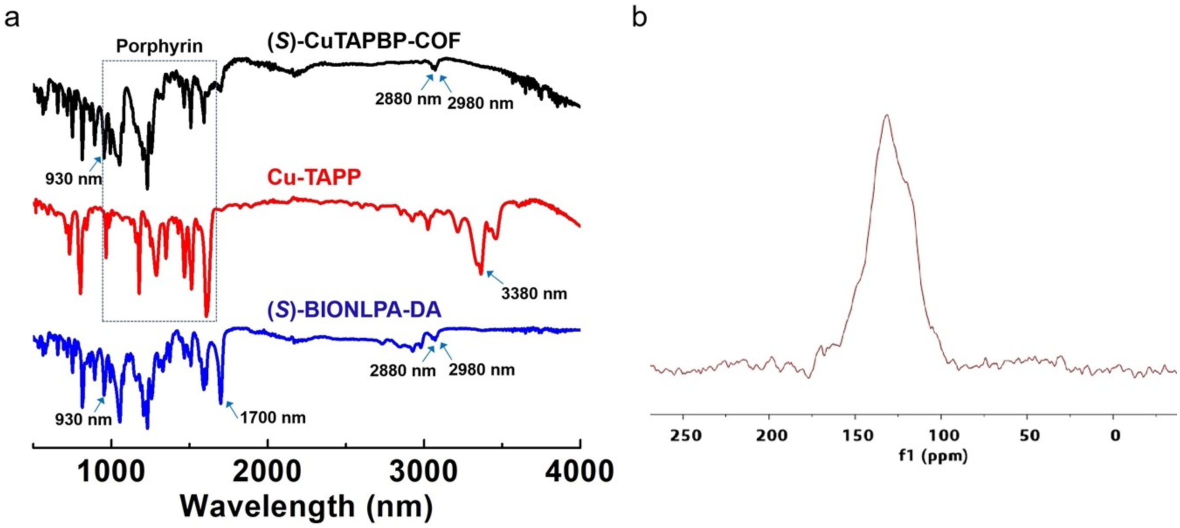

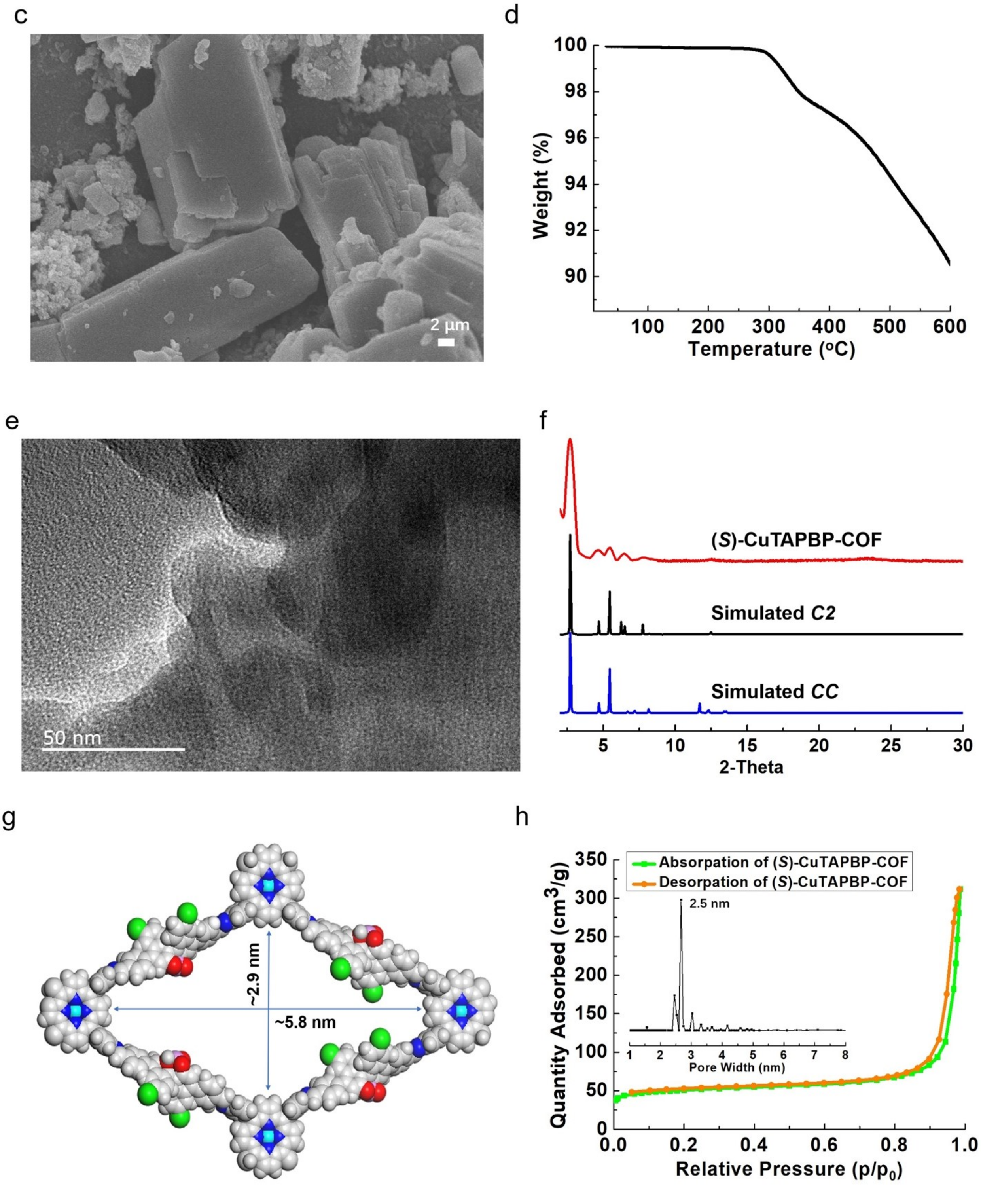
c
2 µm
d
100
98
96
94
92
90
Weight (%)
100
200
300
400
500
600
Temperature (°C)
e
50 nm
f
(S)-CuTAPBP-COF
Simulated C2
Simulated CC
5
10
15
20
25
30
2-Theta
g
~2.9 nm
~5.8 nm
h
Absorpation of (S)-CuTAPBP-COF
Desorpation of (S)-CuTAPBP-COF
2.5 nm
Pore Width (nm)
Quantity Adsorbed (cm³/g)
Relative Pressure (p/p₀)
0.0
0.2
0.4
0.6
0.8
1.0

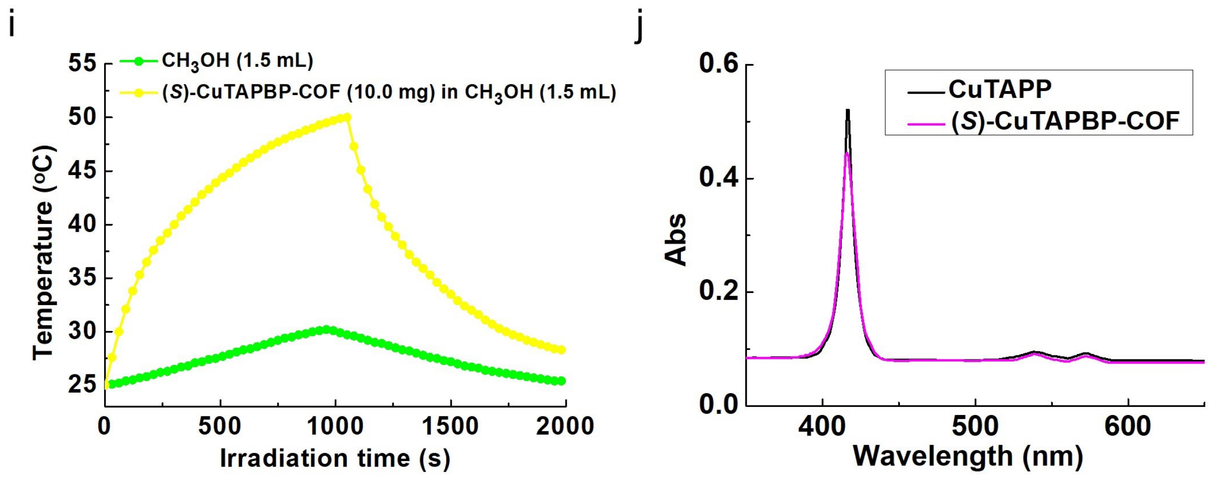

Fig. S11 Characterization of (*S*)-**CuTAPBP-COF**. (a) IR spectra of (*S*)-**CuTAPBP-COF** and its monomers. The IR spectrum of (*S*)-**CuTAPBP-COF** indicated that the characteristic C=O (1700 cm^{-1}) stretching vibration of (*S*)-BINOLPA-DA and N-H (3380 cm^{-1}) stretching band of Cu-TAPP were absent after reaction. Meanwhile stretching vibration bands attributed to P=O linkages were observed at 930 cm^{-1} and characteristic porphyrin stretching vibrations indicated that both the phosphate and porphyrin units existed in CCOF. Elemental Analysis (%) calcd for $C_{112}H_{62}N_8P_2Cu$: C, 85.06; N, 7.09; H, 7.85; found (%): C, 85.03; N, 7.01; H, 7.92. The The P content is 1.03 wt% (calcd, 1.06 wt%) and Cu content is 1.08 wt% (calcd, 1.09 wt%) as determined by ICP-AES. (b) ^{13}C CP-MAS NMR spectrum of (*S*)-**CuTAPBP-COF**. The characteristic resonances in a range of 150-115 ppm are associated with the C=N and BINOL units in CCOF; the signals at 160 ppm are assigned to porphyrin unit. (c) SEM image of (*S*)-**CuTAPBP-COF**. (d) TGA trace of (*S*)-**CuTAPBP-COF**. (e) HRTEM image of (*S*)-**CuTAPBP-COF**. (f) Measured and simulated PXRD patterns for **(***S*)-**CuTAPBP-COF**. Compared to the pattern generated from the *Cc* space group (blue line), (*S*)-**CuTAPBP-COF** unequivocally crystallizes in the C_2 space group. (g) Crystal structure of (*S*)-**CuTAPBP-COF**. (h) N_2 adsorption isotherm of (*S*)-**CuTAPBP-COF** at 77 K. Its N_2 absorption amount at 77 K is 311.5 cm^3 g^{-1}, the corresponding surface area calculated on basis of the BET model is 988.6 m^2 g^{-1}. Its pore width distribution based on nonlocal density functional theory (NLDFT) is inserted. (i) Photothermal behavior of (*S*)-**CuTAPBP-COF** (10.0 mg) in CH_3OH (1.5 mL). The temperature increase (ΔT) is 25 °C. (j) UV-vis spectra of

(*S*)-**CuTAPBP-COF** and Cu-TAPP monomer.

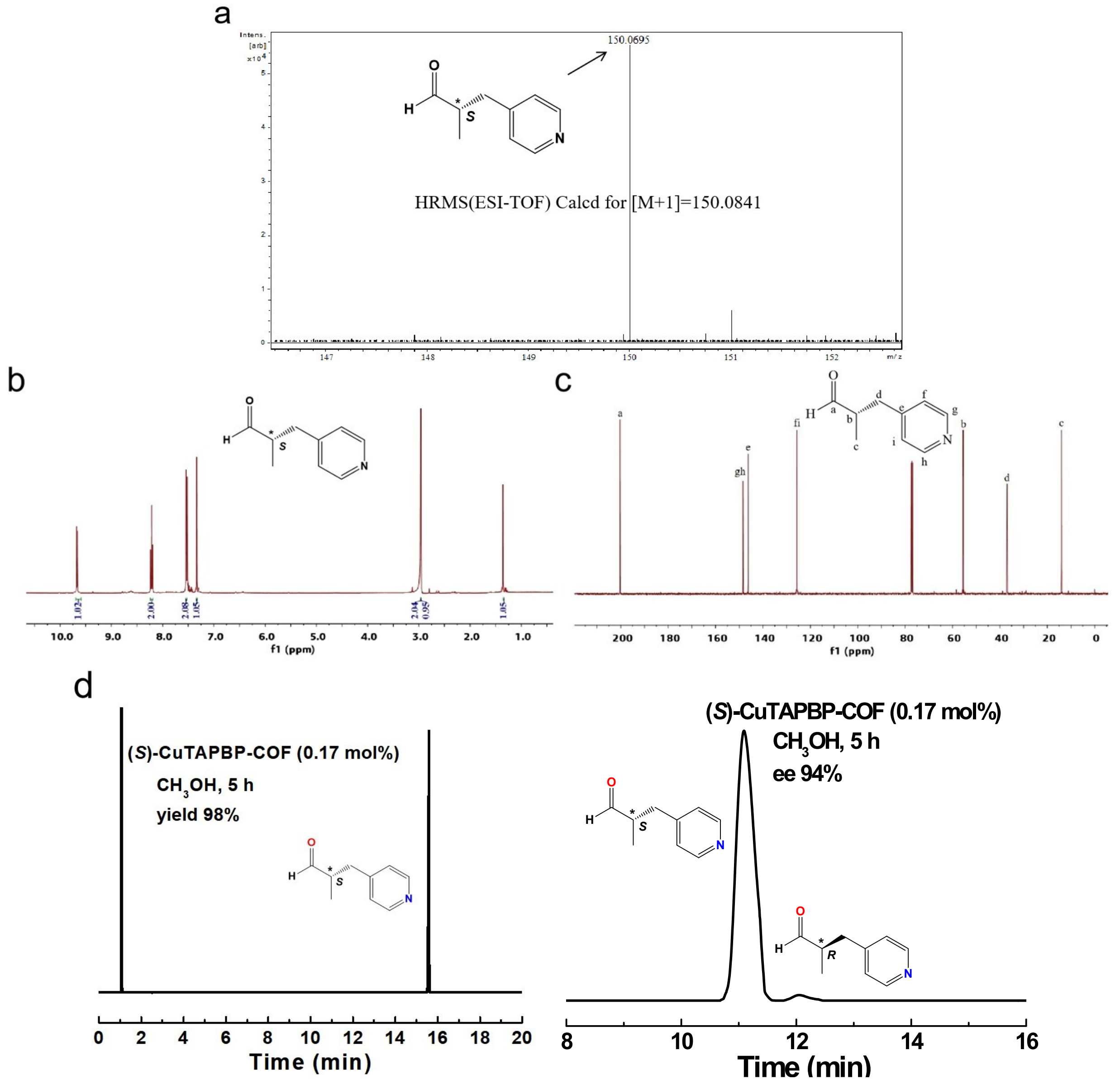

Fig. S12 Characterization of (*S*)-**MPP**. (a) MS spectrum of (*S*)-**MPP**. (b) ^{1}H NMR spectrum of (*S*)-**MPP**. (c) ^{13}C NMR spectrum of (*S*)-**MPP**. (d) Yield and enantiomeric excess of the asymmetric *α*-benzylation reaction of propanal with 4-(bromomethyl)pyridine catalyzed by (*S*)-**CuTAPBP-COF**. Yield was determined by the GC measurement on HP-5 column, and enantiomeric excess was determined by HPLC with a Chiralcel OD-H column (95 : 5 = *n*-hexane : isopropanol, 1.0 mL min^{-1}, 254 nm), respectively.

7. Tables S1-S2

Table S1. The structure model of (*R*)-**CuTAPBP-COF** with C_2 mode.

(*R*)-**CuTAPBP-COF** Space group: C_2<br>a = 66.38 Å, b = 37.65 Å, c = 14.61 Å<br>α = 90.0°, β = 104.5°, γ = 90.0°			
Atom	x	y	z
C1	0.27378	0.29307	0.74342
C2	0.29347	0.28244	0.79806
C3	0.31156	0.29941	0.78815
C4	0.31044	0.32773	0.72438
C5	0.29085	0.33837	0.66947
C6	0.27266	0.32122	0.67861
C7	-0.04194	-0.08538	0.59161
C8	-0.05016	-0.07543	0.66751
C9	-0.03789	-0.05506	0.74111
C10	-0.01789	-0.04406	0.73792
C11	-0.00981	-0.05332	0.66076
C12	-0.02202	-0.07428	0.58807
C13	0.26710	-0.07713	0.77715
C14	0.27874	-0.06756	0.71277
C15	0.29804	-0.08388	0.71690
C16	0.30607	-0.11022	0.78488
C17	0.29414	-0.12020	0.84746
C18	0.27495	-0.10386	0.84396
C19	0.14243	0.23256	0.72345
C20	0.12196	0.23216	0.72561
C21	0.11612	0.19679	0.72374
N22	0.13241	0.17512	0.72102
C23	0.14851	0.19742	0.72044
C24	0.16847	0.18711	0.71480
C25	0.09572	0.18580	0.72092
C26	0.12313	0.01161	0.72440
C27	0.14364	0.01190	0.72345
C28	0.14936	0.04713	0.72040
N29	0.13300	0.06889	0.72027
C30	0.11691	0.04673	0.72210
C31	0.16930	0.05806	0.71585
C32	0.09642	0.05707	0.71963
C33	0.06880	0.13821	0.71813
C34	0.06897	0.10347	0.71706
C35	0.09016	0.09262	0.71759
N36	0.10241	0.12154	0.71832
C37	0.08991	0.14996	0.71883

C38	0.19570	0.10566	0.71349
C39	0.19552	0.14039	0.71375
C40	0.17436	0.15133	0.71416
N41	0.16207	0.12247	0.71462
C42	0.17472	0.09396	0.71386
Cu43	0.13207	0.12198	0.71656
C44	0.25486	0.27434	0.75536
N45	0.23712	0.27665	0.69203
C46	0.21865	0.25765	0.69764
C47	0.20659	0.24113	0.61631
C48	0.18961	0.21973	0.62109
C49	0.18428	0.21465	0.70736
C50	0.19556	0.23310	0.78780
C51	0.21240	0.25503	0.78236
C52	0.18572	0.03069	0.71453
C53	0.08005	0.02973	0.71899
C54	0.07896	0.21245	0.71966
C55	0.19592	0.01285	0.79772
C56	0.21287	-0.00974	0.79927
C57	0.22010	-0.01481	0.71726
C58	0.20969	0.00270	0.63399
C59	0.19257	0.02483	0.63226
C60	0.06589	0.02077	0.63363
C61	0.04774	0.00182	0.63315
C62	0.04320	-0.00822	0.71866
C63	0.05756	0.00015	0.80389
C64	0.07590	0.01867	0.80419
C65	0.06302	0.21706	0.63688
C66	0.04433	0.23375	0.63998
C67	0.04134	0.24703	0.72528
C68	0.05783	0.24441	0.80737
C69	0.07644	0.22711	0.80458
C70	0.01615	0.28013	0.79001
N71	0.02084	0.25921	0.72741
C72	0.01090	-0.04070	0.65299
N73	0.02348	-0.02366	0.72168
C74	0.24721	-0.05914	0.77946
N75	0.23855	-0.03528	0.71723
C76	-0.03120	0.32636	0.83681
C77	-0.04762	0.30503	0.78583
C78	-0.04302	0.27560	0.73571
C79	-0.02233	0.26748	0.73645
C80	-0.00586	0.28886	0.78681

C81	-0.01051	0.31822	0.83733
H82	0.29478	0.25978	0.85099
H83	0.32742	0.29031	0.83206
H84	0.28960	0.36113	0.61689
H85	0.25691	0.33018	0.63322
H86	-0.05149	-0.10261	0.53232
H87	-0.04415	-0.04732	0.80420
H88	-0.00807	-0.02751	0.79805
H89	-0.01553	-0.08221	0.52577
H90	0.27258	-0.04647	0.65680
H91	0.30745	-0.07587	0.66475
H92	0.30000	-0.14186	0.90216
H93	0.26569	-0.11250	0.89617
H94	0.15090	0.25893	0.72431
H95	0.11337	0.25820	0.72837
H96	0.11480	-0.01472	0.72671
H97	0.15244	-0.01419	0.72512
H98	0.05384	0.15304	0.71834
H99	0.05414	0.08803	0.71598
H100	0.21063	0.09078	0.71306
H101	0.21030	0.15579	0.71363
H102	0.25610	0.25768	0.82168
H103	0.21055	0.24506	0.54545
H104	0.17998	0.20625	0.55461
H105	0.19107	0.23031	0.85782
H106	0.22098	0.27071	0.84716
H107	0.19048	0.01660	0.86515
H108	0.22090	-0.02415	0.86721
H109	0.21511	-0.00095	0.56646
H110	0.18420	0.03810	0.56308
H111	0.06910	0.02894	0.56351
H112	0.03650	-0.00561	0.56319
H113	0.05439	-0.00811	0.87403
H114	0.08749	0.02472	0.87431
H115	0.06522	0.20720	0.56626
H116	0.03134	0.23666	0.57265
H117	0.05613	0.25638	0.87670
H118	0.08954	0.22502	0.87177
H119	0.02903	0.29210	0.84853
H120	0.01570	-0.04627	0.58470
H121	0.23955	-0.06690	0.83825
H122	-0.03465	0.35037	0.87794
H123	-0.05613	0.25807	0.69397

H124	-0.01873	0.24338	0.69601
H125	0.00272	0.33550	0.87904
O126	0.63133	0.47144	0.35045
O127	0.61281	0.43706	0.21459
O128	0.64847	0.43956	0.22367
O129	0.64935	0.41808	0.39561
P130	0.63387	0.43782	0.30323
C131	0.57058	0.41086	0.32217
C132	0.57819	0.37786	0.36391
C133	0.59830	0.36553	0.35952
C134	0.61069	0.38636	0.31395
C135	0.60242	0.41830	0.26983
C136	0.58303	0.43090	0.27722
C137	0.63033	0.37085	0.29773
C138	0.64906	0.38837	0.34052
C139	0.66818	0.37557	0.33061
C140	0.66945	0.34404	0.28179
C141	0.65094	0.32449	0.24345
C142	0.63132	0.33795	0.25200
C143	0.56623	0.35681	0.41055
C144	0.57349	0.32398	0.44921
C145	0.59283	0.31155	0.44219
C146	0.60518	0.33207	0.39830
C147	0.61322	0.31765	0.21650
C148	0.61433	0.28492	0.17327
C149	0.63341	0.27180	0.16441
C150	0.65157	0.29135	0.19931
Cl151	0.55823	0.29849	0.50738
Cl152	0.63465	0.23036	0.11045
H153	0.57745	0.45796	0.24588
H154	0.68309	0.39090	0.36263
H155	0.55039	0.36659	0.41702
H156	0.59863	0.28446	0.47245
H157	0.62101	0.32151	0.39397
H158	0.59748	0.32793	0.22297
H159	0.59958	0.26871	0.14492
H160	0.66704	0.28021	0.19176
O161	0.84262	0.27820	0.72980
O162	0.86215	0.29990	0.87338
O163	0.88091	0.25212	0.80981
O164	0.87657	0.28975	0.68236
P165	0.86808	0.28359	0.77844
C166	0.82741	0.37517	0.78621

C167	0.84019	0.39617	0.74280
C168	0.86029	0.38361	0.73897
C169	0.86741	0.35007	0.77804
C170	0.85476	0.33042	0.82405
C171	0.83478	0.34223	0.82466
C172	0.88958	0.33954	0.78596
C173	0.89290	0.30889	0.73644
C174	0.91303	0.29732	0.73838
C175	0.93049	0.31671	0.78574
C176	0.92756	0.34907	0.83085
C177	0.90698	0.36056	0.83042
C178	0.83337	0.42950	0.70251
C179	0.84617	0.45051	0.66224
C180	0.86601	0.43864	0.66129
C181	0.87301	0.40553	0.69875
C182	0.90431	0.39330	0.87262
C183	0.92146	0.41427	0.91460
C184	0.94155	0.40307	0.91556
C185	0.94459	0.37081	0.87393
Cl186	0.83712	0.49185	0.61212
Cl187	0.96305	0.42985	0.96802
H188	0.82448	0.32453	0.85718
H189	0.91541	0.27168	0.70094
H190	0.81718	0.43959	0.70259
H191	0.87661	0.45605	0.62974
H192	0.88923	0.39624	0.69660
H193	0.88802	0.40289	0.87266
H194	0.91910	0.44073	0.94842
H195	0.96110	0.36210	0.87506
H196	0.36043	0.45311	0.84138
H197	0.33361	0.79623	0.76474

Table S2. The structure model of (*S*)-**CuTAPBP-COF** with C_2 mode.

(*S*)-**CuTAPBP-COF** Space group: C_2<br>a = 66.38 Å, b = 37.65 Å, c = 14.61 Å<br>α = 90.0°, β = 104.5°, γ = 90.0°			
Atom	x	y	z
C1	0.19002	0.28916	0.69923
C2	0.20893	0.27591	0.75558
C3	0.22797	0.28979	0.74760
C4	0.22863	0.31756	0.68408
C5	0.20983	0.33080	0.62749
C6	0.19068	0.31674	0.63470

C7	-0.12436	-0.09090	0.55017
C8	-0.13216	-0.08226	0.62826
C9	-0.12025	-0.06052	0.69962
C10	-0.10107	-0.04689	0.69210
C11	-0.09345	-0.05486	0.61276
C12	-0.10527	-0.07718	0.54230
C13	0.18523	-0.07209	0.73336
C14	0.19553	-0.05949	0.66662
C15	0.21562	-0.07179	0.66755
C16	0.22580	-0.09703	0.73461
C17	0.21524	-0.11006	0.79957
C18	0.19526	-0.09774	0.79927
C19	0.05830	0.23249	0.67439
C20	0.03783	0.23209	0.67655
C21	0.03199	0.19672	0.67468
N22	0.04828	0.17506	0.67196
C23	0.06437	0.19736	0.67138
C24	0.08433	0.18704	0.66574
C25	0.01159	0.18573	0.67186
C26	0.03900	0.01155	0.67534
C27	0.05951	0.01183	0.67439
C28	0.06523	0.04707	0.67134
N29	0.04887	0.06883	0.67121
C30	0.03278	0.04667	0.67304
C31	0.08517	0.05800	0.66679
C32	0.01229	0.05701	0.67057
C33	-0.01533	0.13814	0.66907
C34	-0.01516	0.10341	0.66800
C35	0.00602	0.09255	0.66853
N36	0.01828	0.12148	0.66926
C37	0.00578	0.14990	0.66977
C38	0.11157	0.10560	0.66443
C39	0.11139	0.14033	0.66469
C40	0.09022	0.15126	0.66510
N41	0.07794	0.12240	0.66556
C42	0.09059	0.09390	0.66480
Cu43	0.04794	0.12192	0.66750
C44	0.16987	0.27306	0.70887
N45	0.15256	0.27976	0.64097
C46	0.13466	0.25786	0.64832
C47	0.12274	0.24087	0.56743
C48	0.10626	0.21845	0.57317
C49	0.10127	0.21281	0.65998

C50	0.11236	0.23168	0.74002
C51	0.12871	0.25463	0.73363
C52	0.10159	0.03063	0.66547
C53	-0.00389	0.02911	0.67015
C54	-0.00517	0.21238	0.67060
C55	0.11179	0.01279	0.74866
C56	0.12874	-0.00980	0.75021
C57	0.13597	-0.01488	0.66820
C58	0.12556	0.00264	0.58493
C59	0.10844	0.02476	0.58320
C60	-0.01814	0.02057	0.58469
C61	-0.03648	0.00217	0.58399
C62	-0.04112	-0.00773	0.66938
C63	-0.02668	0.00021	0.75471
C64	-0.00815	0.01817	0.75522
C65	-0.02111	0.21700	0.58782
C66	-0.03980	0.23369	0.59092
C67	-0.04279	0.24696	0.67622
C68	-0.02630	0.24435	0.75831
C69	-0.00769	0.22705	0.75552
C70	-0.06695	0.27727	0.73586
N71	-0.06489	0.25550	0.66622
C72	-0.07284	-0.04053	0.60395
N73	-0.06073	-0.02377	0.67873
C74	0.16348	-0.05947	0.73035
N75	0.15471	-0.03525	0.66379
C76	-0.11103	0.33369	0.78806
C77	-0.12867	0.31486	0.73777
C78	-0.12588	0.28419	0.68775
C79	-0.10580	0.27239	0.68790
C80	-0.08812	0.29126	0.73756
C81	-0.09096	0.32187	0.78799
O82	-0.21521	-0.19078	0.61197
O83	-0.19590	-0.19459	0.73299
O84	-0.16712	-0.20071	0.65619
O85	-0.18837	-0.16982	0.53529
P86	-0.18933	-0.19237	0.63129
C87	-0.25079	-0.16738	0.67721
C88	-0.24961	-0.13316	0.63800
C89	-0.23018	-0.12022	0.62602
C90	-0.21211	-0.14165	0.65278
C91	-0.21354	-0.17490	0.69501
C92	-0.23274	-0.18801	0.70350

C93	-0.19135	-0.12585	0.65283
C94	-0.18034	-0.14157	0.59249
C95	-0.16102	-0.12833	0.58662
C96	-0.15244	-0.09805	0.63693
C97	-0.16389	-0.08020	0.69334
C98	-0.18349	-0.09414	0.70080
C99	-0.26743	-0.11146	0.60988
C100	-0.26613	-0.07749	0.57383
C101	-0.24703	-0.06455	0.56501
C102	-0.22924	-0.08564	0.59032
C103	-0.19496	-0.07545	0.75435
C104	-0.18735	-0.04383	0.79987
C105	-0.16822	-0.03026	0.79313
C106	-0.15659	-0.04822	0.74023
Cl107	-0.28868	-0.05121	0.53893
Cl108	-0.15885	0.00978	0.85018
H109	0.20881	0.25365	0.80833
H110	0.24316	0.27859	0.79288
H111	0.21002	0.35315	0.57509
H112	0.17560	0.32778	0.58795
H113	-0.13360	-0.10919	0.49264
H114	-0.12615	-0.05379	0.76443
H115	-0.09154	-0.02925	0.75045
H116	-0.09913	-0.08408	0.47826
H117	0.18762	-0.03921	0.61134
H118	0.22391	-0.06134	0.61350
H119	0.22288	-0.13096	0.85363
H120	0.18715	-0.10879	0.85334
H121	0.06677	0.25887	0.67525
H122	0.02924	0.25814	0.67931
H123	0.03066	-0.01478	0.67765
H124	0.06831	-0.01426	0.67606
H125	-0.03030	0.15297	0.66928
H126	-0.02999	0.08797	0.66692
H127	0.12650	0.09072	0.66400
H128	0.12617	0.15572	0.66457
H129	0.16907	0.25561	0.77163
H130	0.12643	0.24524	0.49614
H131	0.09675	0.20460	0.50703
H132	0.10812	0.22843	0.81049
H133	0.13711	0.27062	0.79812
H134	0.10635	0.01654	0.81609
H135	0.13676	-0.02421	0.81815

H136	0.13098	-0.00101	0.51740
H137	0.10007	0.03804	0.51402
H138	-0.01485	0.02865	0.51465
H139	-0.04780	-0.00493	0.51394
H140	-0.02994	-0.00796	0.82475
H141	0.00349	0.02389	0.82541
H142	-0.01891	0.20713	0.51720
H143	-0.05279	0.23660	0.52359
H144	-0.02800	0.25632	0.82764
H145	0.00541	0.22496	0.82271
H146	-0.05270	0.28518	0.79374
H147	-0.06741	-0.04359	0.53625
H148	0.15447	-0.06971	0.78191
H149	-0.11301	0.35865	0.82910
H150	-0.13999	0.26864	0.64657
H151	-0.10369	0.24732	0.64754
H152	-0.07675	0.33715	0.82914
H153	-0.23352	-0.21597	0.73236
H154	-0.15197	-0.14227	0.54021
H155	-0.28317	-0.12164	0.61651
H156	-0.24589	-0.03656	0.53695
H157	-0.21381	-0.07462	0.58187
H158	-0.21062	-0.08613	0.76079
H159	-0.19679	-0.02892	0.84290
H160	-0.14102	-0.03671	0.73521
O161	0.78802	0.41813	0.74937
O162	0.78899	0.40980	0.62142
O163	0.82633	0.42365	0.66285
O164	0.82534	0.40585	0.81111
P165	0.80951	0.41793	0.70947
C166	0.74707	0.38874	0.73600
C167	0.75595	0.35969	0.79425
C168	0.77674	0.34858	0.79804
C169	0.78853	0.36670	0.74406
C170	0.77906	0.39450	0.68397
C171	0.75896	0.40613	0.68303
C172	0.80908	0.35164	0.73788
C173	0.82679	0.37294	0.77242
C174	0.84665	0.36092	0.77099
C175	0.84971	0.32675	0.73948
C176	0.83228	0.30378	0.71034
C177	0.81190	0.31631	0.71007
C178	0.74461	0.34146	0.84943

C179	0.75315	0.31234	0.90455
C180	0.77319	0.30091	0.90576
C181	0.78493	0.31884	0.85356
C182	0.79490	0.29282	0.68412
C183	0.79781	0.25784	0.65868
C184	0.81763	0.24558	0.65842
C185	0.83473	0.26830	0.68417
Cl186	0.73861	0.29033	0.97296
Cl187	0.82115	0.20133	0.62695
H188	0.75232	0.43018	0.63815
H189	0.86069	0.37909	0.79578
H190	0.72821	0.35048	0.84935
H191	0.78006	0.27676	0.94962
H192	0.80134	0.30919	0.85614
H193	0.77858	0.30234	0.68379
H194	0.78394	0.23906	0.63792
H195	0.85083	0.25785	0.68386
H196	-0.21951	-0.17085	0.65974
H197	0.79037	0.43587	0.81183

8. References

[1] J. Metz, O. Schneider and M. Hanack, *Inorg. Chem*., 1984, **23**, 1065-1071.

[2] C. Valente, E. Choi, M. E. Belowich, C. J. Doonan, Q. Li, T. B. Gasa, Y. Y. Botros, O. M. Yaghi and J. F. Stoddart, *Chem. Commun*., 2010, **46**, 4911-4913.

[3] (a) A. R. Brown, W-H. Kuo and E. N. Jacobsen, *J. Am. Chem. Soc.*, 2010, **132**, 9286-9288. (b) A. L. Berger, K. Donabauer and B. König, *Chem. Sci.*, 2018, **9**, 7230-7235. (c) D. T. Cohen, K. A. Scheidt, *Chem. Sci.*, 2012, **3**, 53-57. (d) M. Rueping, A. Kuenkel and I. Atodiresei, *Chem. Soc. Rev.*, 2011, **40**, 4539-4549.
